# Bipartite structure of the inactive mouse X chromosome

**DOI:** 10.1186/s13059-015-0728-8

**Published:** 2015-08-07

**Authors:** Xinxian Deng, Wenxiu Ma, Vijay Ramani, Andrew Hill, Fan Yang, Ferhat Ay, Joel B. Berletch, Carl Anthony Blau, Jay Shendure, Zhijun Duan, William S. Noble, Christine M. Disteche

**Affiliations:** Department of Pathology, University of Washington, Seattle, Washington USA; Department of Genome Sciences, University of Washington, Seattle, Washington USA; Institute for Stem Cell and Regenerative Medicine, University of Washington, Seattle, Washington USA; Division of Hematology, University of Washington, Seattle, Washington USA; Department of Computer Science and Engineering, University of Washington, Seattle, Washington USA; Department of Medicine, University of Washington, Seattle, Washington USA

## Abstract

**Background:**

In mammals, one of the female X chromosomes and all imprinted genes are expressed exclusively from a single allele in somatic cells. To evaluate structural changes associated with allelic silencing, we have applied a recently developed Hi-C assay that uses DNase I for chromatin fragmentation to mouse F1 hybrid systems.

**Results:**

We find radically different conformations for the two female mouse X chromosomes. The inactive X has two superdomains of frequent intrachromosomal contacts separated by a boundary region. Comparison with the recently reported two-superdomain structure of the human inactive X shows that the genomic content of the superdomains differs between species, but part of the boundary region is conserved and located near the *Dxz4*/*DXZ4* locus. In mouse, the boundary region also contains a minisatellite, *Ds-TR*, and both *Dxz4* and *Ds-TR* appear to be anchored to the nucleolus. Genes that escape X inactivation do not cluster but are located near the periphery of the 3D structure, as are regions enriched in CTCF or RNA polymerase. Fewer short-range intrachromosomal contacts are detected for the inactive alleles of genes subject to X inactivation compared with the active alleles and with genes that escape X inactivation. This pattern is also evident for imprinted genes, in which more chromatin contacts are detected for the expressed allele.

**Conclusions:**

By applying a novel Hi-C method to map allelic chromatin contacts, we discover a specific bipartite organization of the mouse inactive X chromosome that probably plays an important role in maintenance of gene silencing.

**Electronic supplementary material:**

The online version of this article (doi:10.1186/s13059-015-0728-8) contains supplementary material, which is available to authorized users.

## Background

Chromosomes occupy specific territories within the nucleus [1]. In diploid cells, homologous chromosomes occupy separate territories, but expression from genes located on either the paternal or maternal homolog is usually similar. In contrast, X-linked genes are subject to silencing by X chromosome inactivation (XCI) on one of the two homologs in female somatic cells [2], and a subset of autosomal genes are subject to imprinting and expressed from either the paternal or maternal allele [3]. These exceptional genomic regions thus exhibit radically different expression levels of each allele. At the same time, allele-specific chromatin conformation/contact differences are observed at these regions [4–7]. The inactive X chromosome (Xi) becomes highly condensed compared with the active X (Xa) and forms the Barr body, often visible as a dense region within the nucleus [8]. The heterochromatization of one of the X chromosomes in female somatic cells is initiated by the long non-coding RNA (lncRNA) *Xist* that coats the Xi in early embryogenesis and silences transcription by recruiting specific proteins that put in place repressive histone modifications such as tri-methylation of histone H3K27, ubiquitination of histone H2AK119, and de-acetylation [9–12]. Additional layers of silencing involve DNA methylation at the CpG islands of X-linked genes and late replication [13]. Some genes, representing about 10–15 % of X-linked genes in human and 3–5 % in mouse, escape XCI and thus remain expressed from both alleles [14–16]. In addition to becoming condensed, the Xi occupies a particular nuclear compartment near the nuclear membrane or the nucleolus [17, 18]. Imprinted genomic regions also undergo epigenetic and conformational changes associated with silencing of one allele [3]. Such conformation changes are considered to involve the formation of loops that bring enhancers and promoters together at the expressed allele [6, 7].

Little is known about the three-dimensional (3D) structure of the X chromosomes and of alleles at the imprinted regions. Previous studies proposed that the Xi condenses around a core of LINE1 (L1) repetitive elements [19], with genes located in the outer layer and escape genes in the most outer layer [20]. Limited analyses of chromatin contacts at specific regions of the mouse X chromosome have been done using chromatin conformation capture approaches such as 4C and 5C [4, 5]. Approaches to visualize the 3D configuration of the whole nucleus and of entire chromosomes include Hi-C, a method to identify chromatin contacts within chromosomes (intrachromosomal) or between chromosomes (interchromosomal). Topologically associated domains (TADs) representing domains (median size 800 kb) of enhanced intrachromosomal contacts have been defined along the human and mouse genomes by Hi-C [6, 21, 22]. TADs are separated by boundary regions often enriched in CTCF [22], and disruption of a boundary can affect adjacent TADs, supporting a functional role in 3D organization [4].

We have previously reported a novel type of Hi-C method, DNase Hi-C, which uses DNase I rather than restriction enzymes for chromatin fragmentation, leading to improved efficiency and mappability compared with Hi-C methods based on restriction enzyme digestion [23]. By combining our original DNase Hi-C protocol with nuclear ligation [24], we have now implemented an “in situ” version of DNase Hi-C, which is dramatically simplified and much easier to use. As observed with in situ Hi-C [6], this updated protocol also reduces the frequency of spurious contacts due to random ligation. Here, we used both DNase Hi-C and its in situ extension to obtain an allelic 3D view of the mouse genome, including the X chromosomes and imprinted regions in vitro and in vivo.

To discriminate between alleles, we employed F1 mouse hybrid systems that we previously developed, based on single nucleotide polymorphisms (SNPs) in conjunction with skewed XCI [15, 25, 26]. To maximize the number of SNPs that could be used for allele discrimination, C57BL/6J (BL6) female mice were bred to *Mus spretus* males. These two mouse species differ by SNPs with a frequency of 1/70–96 bp, depending on the chromosome. The parent BL6 mice had either an *Hprt* mutation in order to skew XCI (to the BL6 X) in a cell line (Patski) [27], or an *Xist* mutation to skew XCI (to the *spretus* X) in mouse tissues [25]. Imprinted regions can also be examined using these F1 hybrid systems in which the paternal *spretus* and maternal BL6 alleles can be identified. Allelic gene expression measurements by RNA-seq and quantitative RT-PCR previously verified complete XCI skewing and mono-allelic expression of imprinted genes in the F1 hybrid systems [15, 25, 26].

By applying our DNase Hi-C approach [23] as well as the new adapted in situ DNase Hi-C protocol to the mouse F1 hybrid systems described above, we demonstrate that the mouse Xi condenses in a bipartite structure both in the Patski cell line and in mouse brain, representing the first such analysis in vivo. The genomic content of the two superdomains differs between mouse and human where a similar structure of the Xi was also recently reported by Hi-C in cultured human cells [6]. However, the boundary region between the two superdomains is partially conserved and contains elements that bind CTCF and nucleolar proteins. We also show that chromatin contacts differ between loci on the paternal and maternal alleles at imprinted genes as well as between genes that escape XCI and genes subject to XCI, suggesting a functional link between chromatin contacts and transcription.

## Results

### The inactive mouse X chromosome forms a bipartite structure in cultured cells and tissue

We used DNase Hi-C and a modified in situ DNase Hi-C (see details in “Materials and methods”) to obtain allele-specific maps of intrachromosomal contacts on the mouse X chromosomes and autosomes. While our published DNase Hi-C [23], like in situ Hi-C [6], markedly reduces cellular input requirements, the protocol also requires a time-consuming agarose gel proximity ligation. Inspired by single cell Hi-C [28] and in situ Hi-C [6], we simplified our protocol by carrying out proximity ligation within intact nuclei instead of in solid agarose gel. Our updated protocol, termed in situ DNase Hi-C, requires only 2–3 days instead of 6–7 days to generate a library, with considerably less hands-on time and lower costs than the original DNase Hi-C.

Data were obtained from two biological replicates of Patski fibroblast cells using in situ DNase Hi-C. The Patski cell line in which the Xi is from BL6 was originally derived from the kidney of an 18 days postcoitum F1 female embryo obtained by mating a BL6 female with an *Hprt* mutation to a *spretus* male and growing cells in hypoxanthine-aminopterin-thymidine (HAT) medium [27]. Chromosome analyses confirmed a near-diploid karyotype with two X chromosomes. Importantly, we also obtained Hi-C data in vivo by applying DNase Hi-C and in situ DNase Hi-C to a whole brain specimen from an F1 adult female mouse derived from a cross between a BL6 female with an *Xist* mutation and a *spretus* male, in which the Xi is from *spretus* [25]. To identify reads that map to each parental genome in F1 mice, a “pseudo-*spretus*” genome was assembled by substituting known SNPs between BL6 and *spretus* into the BL6 mm9 reference genome as previously described [15]. SNPs were obtained from the Sanger Institute (SNP database Nov/2011 version) and from in-house analysis [26]. After aligning reads separately to the BL6 and to the pseudo-*spretus* genomes we segregated all high-quality uniquely mapped reads (MAPQ ≥30) into three categories: (1) BL6-SNP reads containing only BL6-specific SNP(s); (2) *spretus*-SNP reads containing only *spretus*-specific SNP(s); (3) reads that do not contain valid SNPs. Additional file 1 summarizes the total number of mapped reads and allele-specific reads for each library, and Additional file 2 lists the number of intrachromosomal and interchromosomal contacts, as well as the number of contacts in relation to distance (from close range to ultra-long range) obtained for each experiment. About one-third of intrachromosomal contacts are >100 kb apart, which accounts for TADs and higher scale chromatin organization (Additional file 2).

**Fig. 1:**
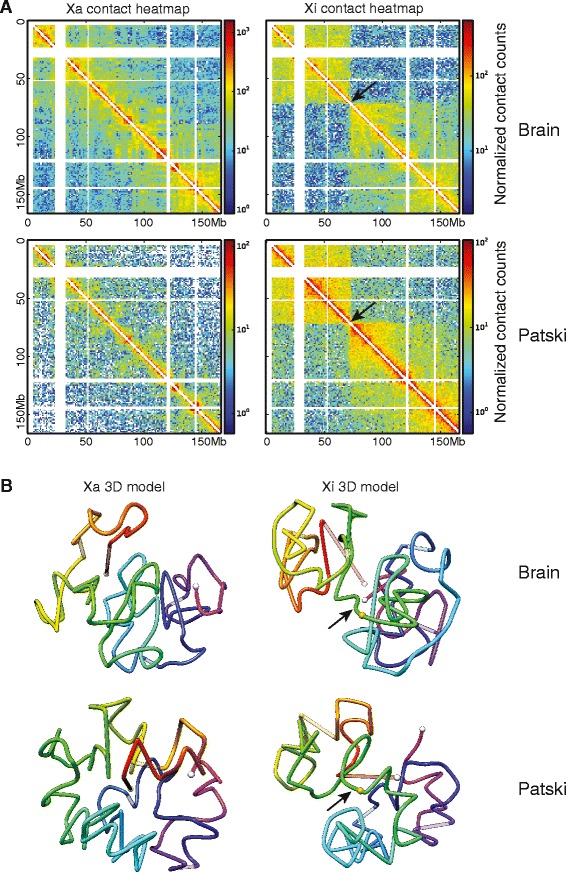
Bipartite structure of the inactive X chromosome in mouse F1 brain and Patski cells. **a** Allelic intrachromosomal chromatin contact heatmaps of the Xa and Xi based on SNP reads at 1 Mb resolution obtained by DNase Hi-C and in situ DNase Hi-C in female F1 brain (*spretus* Xi) and in Patski cells (BL6 Xi). **b** 3D models of the Xa and Xi built on contact frequency at 1 Mb resolution. *White dots* represent chromosome ends; lines are colored from *red* to *purple* in the direction from centromere to telomere; unmappable regions (corresponding to the *white strips* in the heatmaps) are set at 75 % transparency; the *arrow* indicates the hinge region of transition between the two condensed superdomains; the *orange dot* indicates the position of *Dxz4*

The contact maps obtained for each homologous chromosome at 1 Mb resolution by DNase Hi-C or in situ DNase Hi-C in F1 brain and Patski cells show a striking bipartite structure for the Xi, which is very different from that of the Xa (Fig. 1a). In contrast, homologous autosomes appear to have similar structures (Additional file 3). While the Xa forms small contact domains similar to those found on autosomes and representing TADs, the Xi is less topologically organized and shows a high frequency of distant chromosomal contacts within each of two large superdomains (Fig. 1a). Finer-scale analyses at 100 kb resolution for a region around the *Xist* domain (chrX:98.5–103.5 Mb) also showed less defined TADs on the Xi versus the Xa (Additional file 4). Allelic TAD calling at 40 kb was not possible due to insufficient sequence depth. However, combining data from both X chromosomes yielded 102 TADs in F1 brain and 61 TADs in Patski cells, similar to a previous study in cultured fibroblasts [12]. Many of these TADs would be contributed by the Xa.

The two superdomains on the Xi are separated by a boundary region centering at position 72.8–72.9 Mb (mm9) in F1 brain and in Patski cells (Fig. 1a; Additional file 5). These coordinates represent a region with no contact (or the least number of contacts) between superdomains in contact maps at 100 kb and 40 kb resolution (Additional file 5). Hereafter, we refer to superdomain 1 (approximate size 73 Mb) for the domain adjacent to the centromere and superdomain 2 (approximate size 94 Mb) for the distal domain. Within each superdomain we observed a high frequency of contacts (intra-superdomain) compared with the frequency of contacts between superdomains (inter-superdomain) (Table 1). A bipartite index score was calculated as the ratio between the frequency of intra-superdomain to inter-superdomain contacts. In both F1 brain and Patski cells DNase Hi-C data, the observed bipartite index at the boundary region is significant (*p* = 1.25E-3 and 5.67E-3, respectively) for the Xi, but not significant for the Xa (Table 1; Fig. 1).

**Table 1:** Frequency of contacts within superdomains and between superdomains

Library	Chromosome	Frequency of intra-superdomain 1 contacts^a^	Frequency of intra-superdomain 2 contacts^a^	Frequency of inter-superdomain contacts^a^	Bipartite index^b^	*P* value^c^
Combined-brain	Xa	46.72	55.22	13.80	3.69	0.534
Combined-brain	Xi	32.10	33.42	7.81	4.19	1.25E-03
Combined-Patski	Xa	5.26	6.15	2.32	2.46	0.832
Combined-Patski	Xi	12.03	12.97	3.76	3.32	5.67E-03

^a^The frequency of contacts is calculated as the average number of contacts between two 1 Mb-size windows

^b^The bipartite index represents the ratio between the frequency of intra-superdomain contacts to inter-superdomain contacts

^c^The *p* value is calculated by one-sided Z-test to measure the significance of the observed superdomain boundary compared with randomly shifted boundaries

The contact maps obtained by in situ DNase Hi-C for two biological replicates of Patski cells or by regular or in situ DNase Hi-C for F1 brain at 1 Mb resolution show remarkably similar features between replicates, between methods, and between in vitro and in vivo mouse systems (Additional file 6). These results indicate that condensation of the Xi follows a similar pattern in two very different cell types at different developmental stages (embryonic kidney fibroblasts and whole adult brain). A similar structure was reported in a mouse fibroblast cell line [12]. To increase coverage with reads spanning SNPs, data from biological replicates of Patski cells or F1 brain were combined for further analyses. Despite similarities between systems, the index ratio for the bipartite structure was higher for the Xi in F1 brain than Patski cells, suggesting a more condensed structure of the superdomains in brain (Table 1; Fig. 1a; Additional file 6).

Next, we generated 3D models of the mouse X chromosomes, which allowed us to visualize the bipartite structure of the Xi (Fig. 1b). These models are consistent with a turning point or hinge at the boundary region (hereafter named hinge region) between the superdomains. The 3D coordinates of the telomeric ends of the chromosomes are uncertain due to the presence of unmappable regions at the telomeres, especially at the centromeric end that represents a large genomic region enriched in highly repeated, alpha-satellite DNA [29].

### The Xi superdomains differ between human and mouse but the hinge region is near *DXZ4*/*Dxz4* in both species

**Fig. 2:**
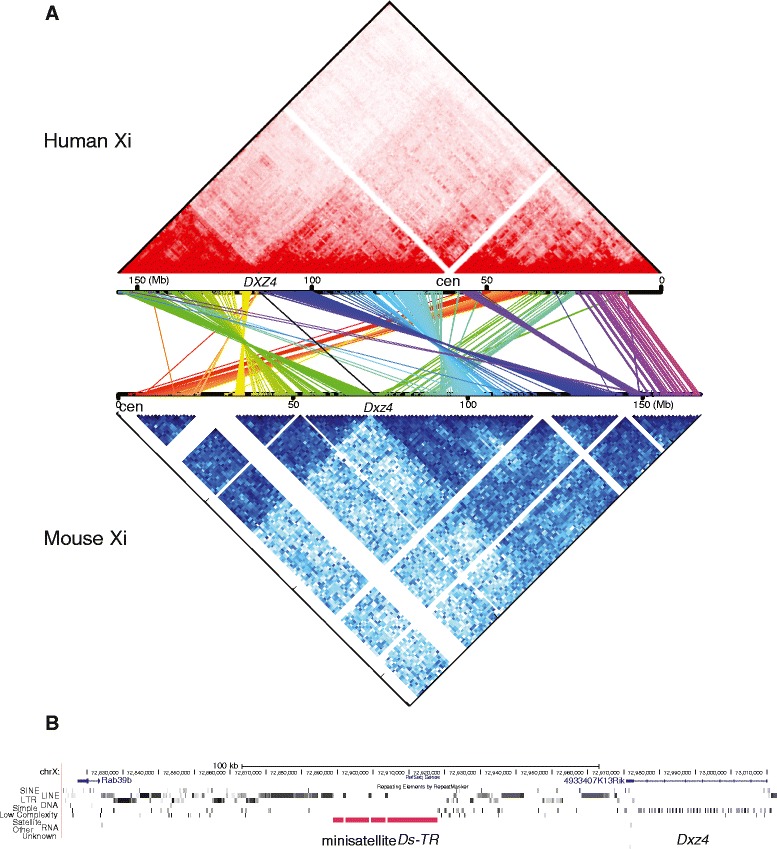
Comparison between superdomains on the Xi in human and mouse. **a** Topological domains on the Xi compared between human and mouse. An allelic contact map of the human Xi generated based on published Hi-C data obtained in human lymphoblastoid cell line GM12878 [6] is shown on *top* (*red*) and aligned to a contact map of the mouse Xi based on our data obtained in mouse F1 brain by DNase Hi-C and in situ DNase Hi-C at *bottom* (*blue*). The Xi maps are compared based on the position of homologous genes between human (hg19) and mouse (mm9) (see “Materials and methods”). The human and mouse X chromosomes were oriented such that the position of *DXZ4*/*Dxz4* and the adjacent *PLS3*/*Pls3* genes are in the same orientation at the right extremity of the hinge region. Each pair of homologous genes is connected by a *colored line* between the contact maps and genes within blocks of conserved regions are indicated in a similar color. Several inversions and transpositions are apparent and the content of the superdomains is only partially conserved. **b** Gene content of the hinge region in mouse (~72.8–72.9 Mb). *Dxz4* is located at one extremity of the hinge region, which also contains the minisatellite *Ds-TR*. The size and location of the hinge region were estimated as described in the text (see also Additional file 5)

To determine whether contact maps were conserved in mammals, we compared topological domains within the mouse Xi with those previously reported in a human lymphoblastoid cell line [6] (Fig. 2a). In human, the two superdomains on the Xi are of unequal size: superdomain 1 (115 Mb) contains the short arm, centromere and proximal long arm, while superdomain 2 (40 Mb) contains the distal long arm. In contrast, the two mouse superdomains are closer in size (72 Mb for superdomain 1 and 94 Mb for superdomain 2). Maps of synteny between human and mouse X chromosomes show that the gene content of the superdomains differs between species. There are several inversions of genomic material between species, indicating that the Xi 3D structure is not conserved. For example, loci included in mouse superdomain 1 are found in separate superdomains in human (Fig. 2a).

Despite differences in the genomic content of the superdomains, the hinge region is partially conserved. In mouse, the end of the hinge region distal to the centromere is near the *Dxz4* locus (nucleotides 72,970,797–73,010,038) that transcribes the lncRNA *4933407K13Rik* (Fig. 2b; Additional file 4). Similarly, one end of the hinge region is near the *DXZ4* locus on the human Xi [6]. The conserved *Dxz4*/*DXZ4* loci represent macrosatellite repeats previously shown to bind CTCF on the Xi in both human and mouse by comparison of male and female samples [30, 31]. Interestingly, the mouse hinge region also contains a 29 kb gamma satellite repeat, *Ds-TR* (downstream inverted tandem repeat; nucleotides 72,888,859–72,917,881) flanked by a potential promoter region [31]. This minisatellite and flanking promoter region are apparently not present in human.

**Fig. 3:**
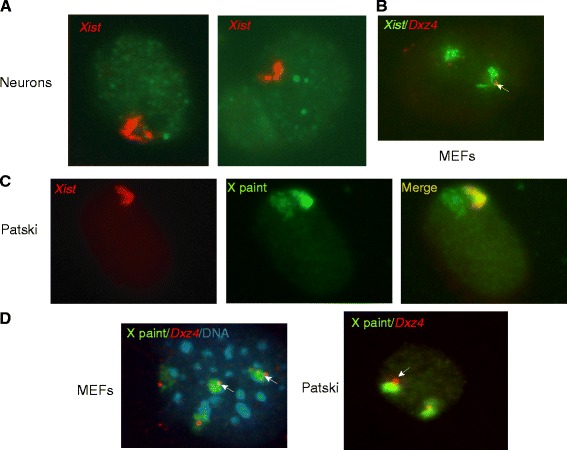
FISH analysis of the mouse Xi. **a** Examples of nuclei after RNA-FISH for *Xist* (*red*) in neuronal cells show a bipartite structure for the Xi, consistent with the 3D structure detected by DNase Hi-C. **b** DNA-FISH for *Dxz4* (*red*) following RNA-FISH for *Xist* (*green*) in MEFs shows that *Dxz4* is located between the two regions coated by *Xist* RNA, consistent with *Dxz4* location at the hinge region. Note that there are two inactive Xs marked by *Xist* RNA clouds in this MEF line. **c** While RNA-FISH for *Xist* (*red*) in Patski cells shows the bipartite structure, DNA-FISH using a mouse X chromosome paint probe (*green*) shows only a condensed structure for the Xi. **d** DNA-FISH using both a mouse X paint (*green*) and *Dxz4* (*red*) shows that *Dxz4* is located at the edge of the condensed Xi in MEFs and Patski cells*.* MEF nucleus is stained with DAPI (*blue*). The *arrows* mark *Dxz4* on the Xi

To determine whether the Xi superdomains and the hinge region could be visualized by microscopy, we performed RNA-fluorescence in situ hybridization (RNA-FISH) for *Xist*, which showed coating of two separate regions on the Xi in a subset of nuclei (<10 %) in female primary neurons, mouse embryonic fibroblasts (MEFs), and Patski cells (Fig. 3a–c). The low frequency of nuclei in which we observed a bipartite structure using *Xist* RNA-FISH may be due to the limitation of two-dimensional FISH and/or to the loss of the 3D structure under the denaturation and hybridization conditions of the FISH procedure. DNA-FISH for *Dxz4* following *Xist* RNA-FISH showed a single signal located between the two *Xist*-coated regions on the Xi, suggesting that these regions represent the superdomains detected by Hi-C (Fig. 3b). DNA-FISH using a whole mouse X chromosome paint together with a probe for *Dxz4* confirmed that *Dxz4* is preferentially located on the outside of the condensed Xi, even though the bipartite structure was not clearly visible using the X paint (Fig. 3d).

### The hinge region on the mouse Xi binds CTCF and associates with the nucleolus

**Fig. 4:**
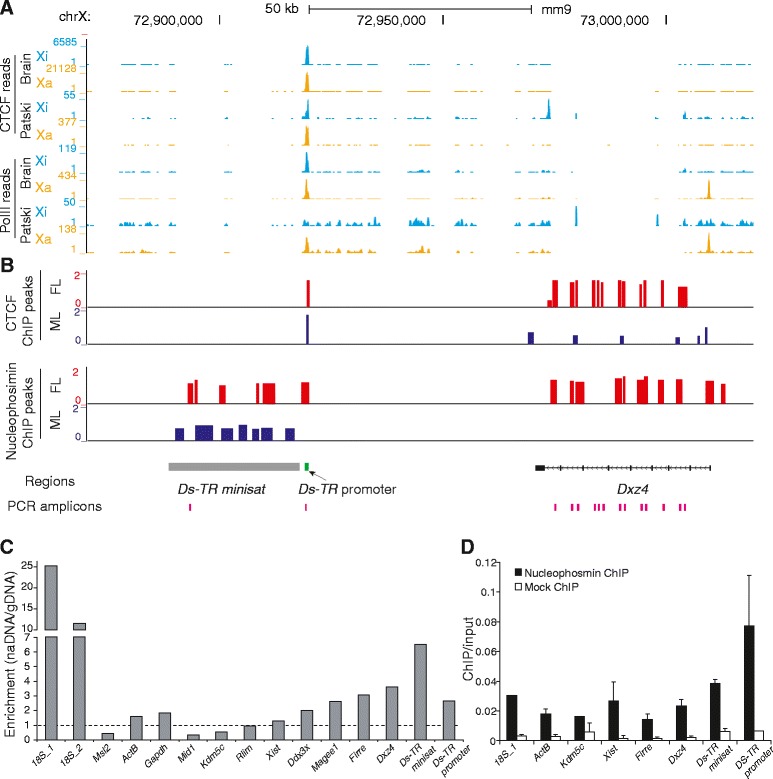
The mouse hinge region binds CTCF and associates with the nucleolus. **a** Allelic profiles of CTCF and PolII binding in F1 brain and Patski cells are shown for the Xi (*blue*) and Xa (*orange*) at the minisatellite *Ds-TR*, its adjacent promoter region, and at *Dxz4*. The *Ds-TR* promoter binds CTCF on the Xa and Xi. No reads were mapped within the minisatellite *Ds-TR* or at *Dxz4* due to low mappability. Different y-axis scales were used for the Xi and Xa in order to show the significant peaks on the Xi, given that there are about threefold more reads at the *Ds-TR* promoter peak region on the Xa compared with the Xi. **b** ChIP-chip analysis for CTCF and nucleophosmin in female (*FL*) and male liver (*ML*). CTCF binds at the *Ds-TR* promoter region in female and male liver, and at *Dxz4* in female but not male liver. Nucleophosmin binds to *Ds-TR*, its promoter, and *Dxz4* in female liver, while in male liver lower binding is present at *Ds-TR*. **c** Enrichment in DNA sequences representing nucleolus-associated domains measured by quantitative PCR in the nucleolus-associated fraction (*naDNA*) versus genomic DNA (*gDNA*) is seen at the minisatellite *Ds-TR*, its promoter and at *Dxz4* in Patski cells. The positions of quantitative PCR amplicons used to measure enrichment at these three regions are indicated in (**b**). Enrichment at control autosomal and X-linked genes is shown. Two primer pairs for different regions of the *18S* ribosomal RNA gene known to be associated with the nucleolus serve as positive controls. The *dashed line* indicates no enrichment (*naDNA*/*gDNA* ratio of 1). **d** Quantitative PCR analysis of chromatin immunoprecipitation (ChIP) for nucleophosmin confirms high enrichment at *Ds-TR* and adjacent promoter. *Error bars* indicate s.e.m.

We previously reported allele-specific profiles of CTCF binding and of RNA polymerase II phosphorylated at serine 5 (hereafter PolII) occupancy obtained by ChIP-seq in F1 brain and Patski cells [15]. Examination of the hinge region in these datasets shows strong CTCF and PolII peaks at the *Ds-TR* promoter (nucleotides 72,919,240–72,919,749) on both the Xi and Xa, especially in F1 brain, suggesting that *Ds-TR* is expressed on both alleles (Fig. 4a). Note that enrichment was always lower on the Xi than the Xa, similar to what is observed for other genes that escape XCI [15]. No CTCF binding was apparent at *Dxz4* due to the low mappability of repeated sequences. However, by ChIP-chip analysis CTCF binding at *Dxz4* was much higher in female than male mouse liver (Fig. 4b), consistent with Xi binding and with previous studies [31, 32]. Except for strong binding at the promoter, there was no evidence of CTCF binding along the minisatellite *Ds-TR* by either ChIP-seq or ChIP-chip (Fig. 4a, b). CTCF motif analysis using FIMO (Find Individual Motif Occurrences) [33] identified three adjacent CTCF binding motifs at the *Ds-TR* promoter location.

Our previous studies have shown that the lncRNA loci *Dxz4* and *Firre* associate with the nucleolus surface when located on the Xi [32], and the Xi is known to visit the nucleolus [18]. To determine whether the hinge region between the two superdomains on the mouse Xi represents a nucleolus-associated domain (NAD), we isolated nucleoli from Patski cells after fixation to capture genomic regions that associate with the nucleolus [34, 35]. Quantitative PCR (qPCR) showed that *Dxz4*, *Ds-TR*, and its promoter were all enriched in the nucleolar fraction, with *Ds-TR* showing the highest enrichment representing a 6.5-fold increase (Fig. 4c). A 12–25-fold increase was seen for a positive control represented by the *18S* ribosomal RNA gene known to associate with the nucleolus (Fig. 4c). The lncRNA loci *Firre* and *Xist* showed a 3.1- and 1.3-fold enrichment, respectively. Three control autosomal genes (*Msl2*, *ActB*, *Gapdh*) and five control X-linked genes (*Mid1*, *Kdm5c*, *Rlim*, *Ddx3x*, *Magee1*) showed low enrichment (0.4–2.6-fold).

We next performed ChIP-chip analysis for nucleophosmin, a protein located at the periphery of the nucleolus. Enrichment was observed at *Dxz4*, *Ds-TR*, and the *Ds-TR* promoter in female liver while in male liver enrichment was seen only at *Ds-TR*, suggesting Xi-specific nucleophosmin binding at specific loci (Fig. 4b). ChIP-qPCR confirmed enrichment in nucleophosmin at these loci especially at the *Ds-TR* promoter (Fig. 4d). Taken together, our results indicate that the hinge region between the two superdomains on the Xi represents an NAD in mouse. Interestingly, the human *DXZ4* locus has also been reported to represent a NAD in HeLa cells [34].

### Distribution of genes, PolII occupancy, CTCF binding and L1 density in relation to the 3D structure of the Xi

**Fig. 5:**
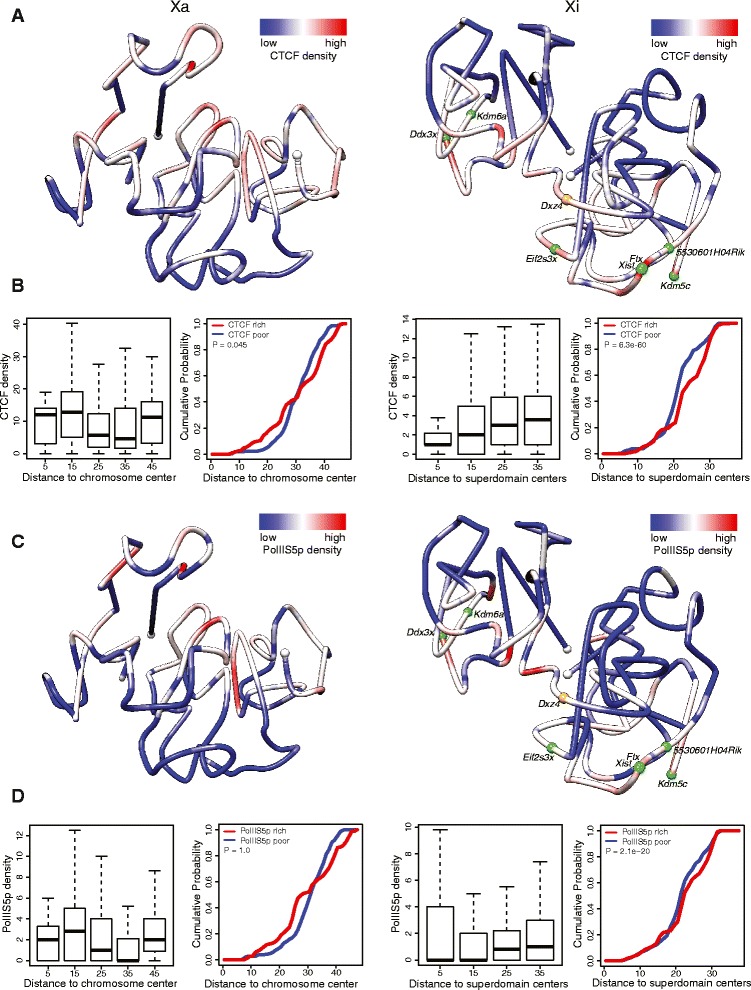
Distribution of CTCF and PolII binding on 3D models of the Xa and Xi. **a** 3D models of the Xa (*left*) and Xi (*right*) at 1 Mb resolution in mouse F1 brain colored to display the density of allelic CTCF binding (*red* indicates more binding). CTCF binding tends to be denser at the periphery of the Xi 3D structure, possibly on one face of the model. *White dots* indicate chromosome ends, *orange dot Dxz4*, *green dots* escape genes. **b** Box plots for the Xa (*left*) and the Xi (*right*) showing allele-specific CTCF-peak density at 1 Mb resolution grouped by the corresponding distances of the 1 Mb regions to the Xa chromosomal center or to the Xi superdomain centers, and empirical cumulative curves of 1 Mb regions binned based on their distance to the Xa chromosomal center or to the Xi superdomain centers for the CTCF-rich (*red line*, top 25 % CTCF-binding regions) and CTCF-poor regions (*blue line*, bottom 25 %). The empirical cumulative density as a function of the distance to the chromosome or superdomain centers for the feature-rich and feature-poor regions were compared using one-side Wilcoxon rank-sum test. **c**, **d** Same analysis for allelic PolII occupancy. Like CTCF, PolII occupancy tends to be higher at the periphery of the Xi 3D structure

**Fig. 6:**
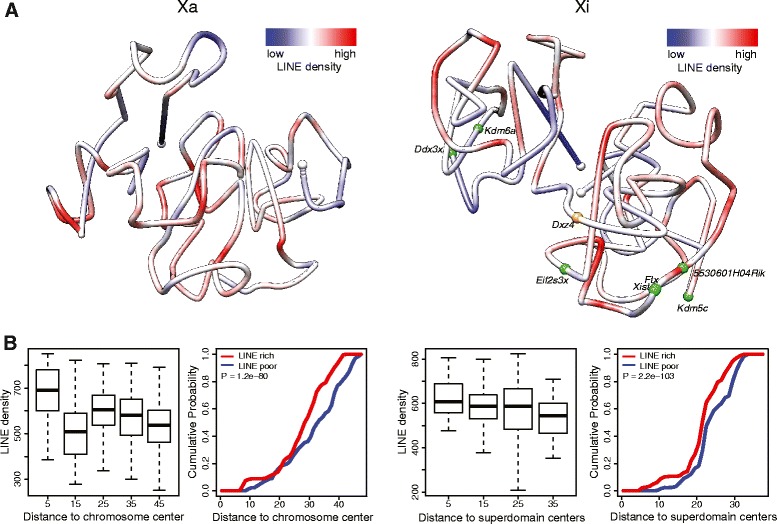
Distribution of L1 elements on 3D models of the Xa and Xi. **a** 3D models of the Xa and Xi at 1 Mb resolution in mouse F1 brain colored to display the density of L1 elements (*red* indicates more L1 elements). L1 elements tend to be located at the inside of the Xi 3D structure. **b** Box plots for the Xa (*left*) and the Xi (*right*) showing L1 density at 1 Mb resolution grouped by the corresponding distances of the 1 Mb regions to the Xa chromosomal center or to the Xi superdomain centers, and empirical cumulative curves of 1 Mb regions binned based on their distance to the Xa chromosomal center or to the Xi superdomain centers for the L1-rich (*red line*, top 25 %) and L1-poor regions (*blue line*, bottom 25 %). The empirical cumulative density as a function of the distance to the chromosome or superdomain centers for the feature-rich and feature-poor regions were compared using one-side Wilcoxon rank-sum test

Allelic distributions of CTCF and PolII datasets [15] were combined with the 3D models of the X chromosomes to visualize the position of regions enriched in CTCF and in active transcription (Fig. 5). Visual inspection of the 3D models of the X chromosomes indicates that CTCF and PolII tend to bind to regions on the outside of the 3D structure of the Xi but not the Xa (Fig. 5a, c). The density of CTCF binding or PolII occupancy in 1 Mb bins along the Xi was positively correlated with the bin distance to the center of each superdomain, confirming significant enrichment in CTCF binding and in active transcription at the periphery of the Xi, but not the Xa (Fig. 5b, d). In contrast, the reverse pattern was observed for regions enriched in L1 elements, which were preferentially located on the inside of the 3D structure (Fig. 6a, b). Note that the density of CTCF binding appears greater on one side of the surface of the Xi 3D structure, possibly representing attachment to the nucleolus surface or to the nuclear membrane (Fig. 5a); however, further studies will be needed to confirm this arrangement. Interestingly, the non-random distribution pattern of regions enriched with CTCF, PolII or L1 elements on the Xi 3D model is more evident in F1 brain compared with Patski cells (data not shown), supporting a more constrained organization of the Xi in brain.

**Fig. 7:**
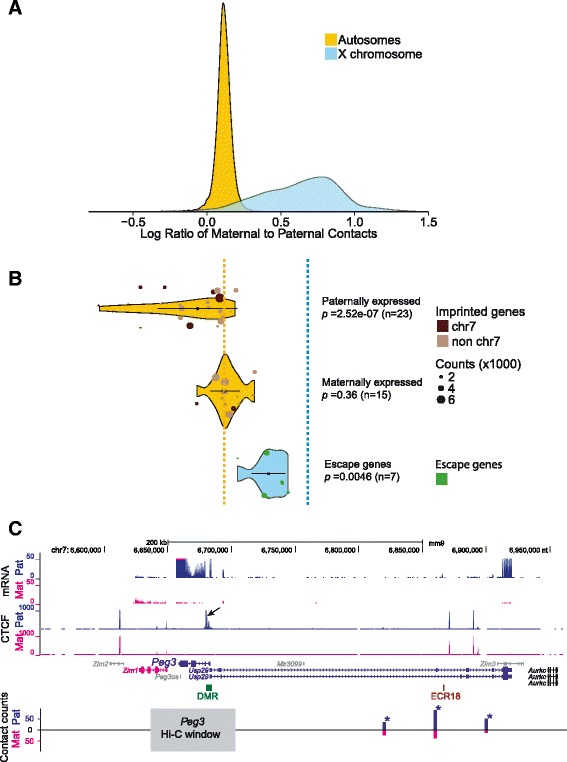
Intrachromosomal contacts at X-linked genes and at imprinted genes. **a** Distribution of maternal-to-paternal allelic contacts at autosomal genes and X-linked genes determined by DNase Hi-C at 40 kb resolution in mouse F1 brain in which the paternal autosomes and the Xi are from *spretus*. Compared with autosomal genes, X-linked genes show high maternal-to-paternal ratios, indicating less frequent contacts at silent genes on the Xi. **b** Violin plots show the distribution of maternal-to-paternal allelic contacts at maternally and paternally imprinted genes and at genes that escape XCI at 40 kb resolution in F1 brain. Compared with other autosomal genes paternally expressed imprinted genes have a lower maternal-to-paternal contact ratio as shown by a long tail. These genes are preferentially located on chromosome 7 and when they are removed from the analysis, the shape of the distribution changes (as shown by a shorter tail in Additional file 8). The chromosomal location of imprinted genes is indicated by *dots* color-coded to indicate the chromosome of origin. The distribution of maternal-to-paternal allelic contacts for genes that escape XCI differs from the rest of X-linked genes, reflecting a higher number of contacts at expressed alleles. *Dotted lines* indicate median ratios of maternal-to-paternal contacts at autosomal and X-linked genes. **c** Significant contacts are detected between the imprinted paternally expressed gene *Peg3* and neighboring regions on the paternal allele. Allelic RNA-seq confirms *Peg3* expression on the paternal allele. Allelic CTCF profiles show binding to the differentially methylated region (*DMR*) adjacent to *Peg3* promoter region only on the paternal allele (*arrow*), presumably facilitating the formation of contacts between the *Peg3* promoter region and the distant enhancer *ECR18* (evolutionarily conserved region 18) [63]. The needle plot of contacts counts between a 40 kb window that overlaps *Peg3* (*grey bar*) and nearby regions shows more interactions on the paternal (*blue*, *Pat*) than the maternal allele (*pink*, *Mat*). Genes with maternal or paternal expression are colored in *pink* or *blue*, non-imprinted genes in *black*, and non-expressed genes in *grey*. Contact regions showing significant allelic biases are marked by *asterisks*

We next determined the position within the 3D structure of the Xi of a subset of seven genes that were previously shown to consistently escape XCI in F1 brain and other tissues as well as Patski cells [15, 26] (Additional file 7). These escape genes were found to be located in the outer layer of the 3D structure of the Xi in F1 brain (*p* = 0.004, Z-test; Fig. 5a, c). We then compared intrachromosomal contacts at autosomal genes (22,874), X-linked genes (975), and the seven escape genes in F1 brain in terms of the ratio of contacts on the maternal chromosome (BL6) to those on the paternal chromosome (*spretus*) (Fig. 7a). Most autosomal genes had a similar number of intrachromosomal contacts on each allele, although there was a slight shift towards maternal contacts, probably due to biased mapping of reads to the reference genome (BL6). In contrast to autosomal genes, X-linked genes, the majority of which are subject to XCI, had more contacts on the Xa than the Xi allele, indicating fewer contacts on the silent copy (Fig. 7a). Genes that escape XCI and are expressed from both alleles showed contact ratios intermediate between autosomal and X-linked genes (*p* = 0.0046, Kolmogorov-Smirnov test; Fig. 7b). Our results show that escape genes have a greater number of specific contacts than inactivated genes on the Xi. Based on a previous 4C study in which contacts among a subset of escape genes were reported in mouse neuronal cells, we also considered potential interactions between specific escape genes [5]. However, we did not detect such specific interactions, probably due to the limited allelic read coverage and resolution in our Hi-C analysis.

### Differential interactions at paternally or maternally imprinted regions

Maternal and paternal alleles of imprinted genes are differentially expressed and thus expected to have differential structure in terms of intrachromosomal contacts [3]. We examined a total of 38 genes imprinted in mouse brain, representing 15 genes expressed on the maternal allele and 23 genes expressed on the paternal allele (Additional file 7). The list of genes considered here was based on a previous study [36] and confirmed by examining our own allelic RNA-seq data in F1 brain (data not shown). Measurements of intrachromosomal contacts in F1 brain in which the maternal allele is from BL6 and the paternal allele from *spretus* showed a higher contact frequency on the expressed allele (Fig. 7b). Examples of significant cis contacts on the expressed allele are shown using needle plots for a 40 kb Hi-C window at the paternally expressed gene *Peg3* and at the maternally expressed gene *Kcnk9* (Fig. 7c; Additional file 8: Figure S5a). When considering all imprinted genes, we confirmed that intrachromosomal interactions were more frequent at the active allele based on violin plots (paternally expressed *p* = 2.5e-7, maternally expressed *p* = 0.36, Kolmogorov-Smirnov test; Fig. 7b). Genes expressed from the paternal allele have an especially high number of contacts, and hence show a long tail toward low maternal-to-paternal contact ratios. A tail toward high maternal-to-paternal contact ratios is not observed for maternally expressed genes, which show a tighter distribution of ratios. Interestingly, almost all genes with high contact number (1000 contacts or more) on the active paternal allele are located on mouse chromosome 7 (Fig. 7b). This is not surprising because chromosome 7 has 9 out of 23 paternally expressed and 2 out of 15 maternally expressed imprinted genes. The distribution of the maternal-to-paternal ratios for all genes on each autosome was similar (data not shown), indicating that the effect seen for imprinted genes on chromosome 7 is unique to paternally expressed genes on this chromosome (Fig. 7b). Accordingly, when removing genes located on chromosome 7 the tail of the distribution for paternally expressed genes is shorter, confirming that paternally expressed genes on chromosome 7 contribute to a high contact frequency (Additional file 8: Figure S5b).

## Discussion

Using DNase Hi-C [23] and a novel in situ DNase Hi-C we discovered that the mouse Xi condenses in two three-dimensionally defined superdomains. We applied our new Hi-C methods to an in vivo system (mouse brain) demonstrating the feasibility and reproducibility of this method for the determination of the structure of individual chromosomes in tissues. Our allele-specific approach provides a comprehensive contact map of paternal and maternal homologous chromosomes and will help better understand tissue-specific and homolog-specific differences in nuclear organization.

Comparisons between published human data [6] and our mouse data revealed surprising differences between superdomains identified in these species. Large differences in the sequence content and organization of the superdomains between species implies that the folding of the Xi may only be partially determined by its sequence. However, the hinge region is partially conserved and located near the *Dxz4*/*DXZ4* macrosatellite locus in both species, which suggests that this locus has a conserved role in terms of organization of the 3D Xi structure. The *Dxz4*/*DXZ4* loci transcribe lncRNAs and bind CTCF on the Xi [30–32], which may facilitate the formation of the two superdomains. lncRNAs have been proposed as key elements of nuclear organization [37]. Expression of *Dxz4* (*4933407K13Rik*) in brain and Patski cells was very low (<1 RPKM (reads per kb of exon per million mapped reads), possibly due to failure to detect small transcripts and/or low mappability of the repeat. Thus, whether the *Dxz4* lncRNA plays a role in the formation of the hinge region is still unclear. Substantial structural differences have been found in mouse versus the primate *Dxz4*/*DXZ4* [31, 38]. The most significant difference is that in human and other primates *DXZ4* is composed of as many as 100 copies of a 3 kb GC-rich repeat, while in mouse *Dxz4* contains about seven repeats ranging in size from 3.8 kb to 5.7 kb that are not particularly GC-rich. Interestingly, we found that the mouse hinge region also contains a novel gamma minisatellite *Ds-TR* not found elsewhere in the mouse genome. *Ds-TR* consists of a palindrome repeat spanning ~30 kb, located ~50 kb downstream of *Dxz4* and absent on the human or rat X chromosomes. Although no RNA-seq reads were observed at *Ds-TR*, probably due to low mappability and/or failure to detect small transcripts, *Ds-TR* is apparently expressed from the Xi as evident by our findings of allelic PolII occupancy at least in F1 brain, in agreement with a previous study that compared transcription in female and male cells [31]. The role of gamma satellites is poorly understood but one such repeat has been shown to prevent the spread of heterochromatin at pericentromeric regions, suggesting that *Ds-TR* could help form a boundary between the two superdomains on the Xi [39]. We speculate that *Dxz4* and *Ds-TR* may function together as chromatin boundaries between the Xi superdomains. However, the molecular mechanisms of formation of a bipartite structure remain to be analyzed to determine whether expression of *Dxz4* and *Ds-TR*, and/or CTCF binding, and/or nucleolus association are independent or related factors in the formation of superdomains on the Xi. Recent studies have reported that the lncRNA *Xist* recruits structural proteins [10–12]. Interestingly, we found that *Xist* RNA-FISH could reveal the Xi bipartite structure, which was not clearly seen by DNA-FISH using an X-paint, suggesting that *Xist* may facilitate contacts within the Xi superdomains.

One important chromosomal organizer protein is the zinc finger protein CTCF often found at the transition between TADs [6, 21, 22]. CTCF-binding at domain boundaries helps anchor chromatin loops and frequently link promoters and enhancers [6]. However, CTCF binding at the hinge region of the Xi is clearly not sufficient to explain the formation of the two superdomains because CTCF binding can be found concentrated elsewhere on the Xi where no superdomains are detected. For example, we and others reported a cluster of CTCF binding specifically on the Xi at the *Firre* locus [32, 40] and CTCF also binds near *Xist* on the mouse Xi [41]. The human homologs of these loci (*XIST*, *FIRRE*) together with *DXZ4* and another lncRNA locus, *LOC550643*, were previously shown to contact each other and to function as anchor regions for superloops on the human Xi [6, 42]. Our datasets did not have sufficient resolution to detect long-range contacts between the corresponding loci in mouse. However, our previous DNA-FISH studies in mouse fibroblasts failed to show association between *Dxz4* and *Firre*, suggesting differences between species [32].

We found that regions enriched in CTCF binding on the Xi tended to be located at the periphery of the 3D structure, suggesting that these loci may serve as attachment sites. Indeed, the mouse and human Xi often occupy specific locations in the nucleus near the lamina or the nucleolus [17, 18]. Based on recent studies of lamina-associated domains (LADs) and NADs, it may be that the nucleolus and nuclear periphery act as “velcro” for heterochromatin including the Xi [43]. An early study proposed that the Barr body represents a looped structure formed by telomeric association with the nuclear membrane [44]. Due to low mappability, we unfortunately could not determine the structure of the telomeric ends of the Xi. However, we found that the *Dxz4*/*Ds-TR* region represents a NAD that binds the nucleolar protein nucleophosim. Thus, the hinge region between superdomains represents a large NAD whose tethering to the nucleolus may govern the formation of a bipartite structure. Whether the hinge region provides flexibility to the Xi 3D structure is unknown. Positioning of the Xi within the nucleus is important for maintenance of its heterochromatic structure. We recently reported that both *Dxz4* and *Firre* associate with the surface of the nucleolus and that *Firre* helps maintain H3K27me3, a repressive histone modification that marks the Xi [32].

Our 3D analyses of the Xi show that genes that escape XCI are located at the periphery of the 3D structure, as previously reported [19]. However, we did not detect specific contacts between these genes, in contrast to a previous 4C study [5], which may be due to the lower resolution of our Hi-C data. Our observation of more short-range contacts on the Xi at genes that escape XCI versus genes subject to XCI may reflect random contacts between inactivated genes on the Xi, while specific interactions would take place at expressed genes. Similarly, a larger number of specific intrachromosomal contacts were also found on the Xa versus the Xi, resulting in more defined topological domains on the Xa, consistent with previous studies [4–6, 12]. These observations do not exclude inactivated genes having many intrachromosomal contacts on the Xi, if those contacts were variable from cell-to-cell and thus not detected by Hi-C done on bulk tissue. Indeed, high-resolution FISH combined with 3C analyses and single cell Hi-C analyses have shown cell-to-cell variability in TAD conformation [28, 45]. The superdomains on the mouse Xi appear less condensed in Patski cells than in F1 brain. Interestingly, our previous studies show a lower density of CTCF sites and more genes that escape XCI in Patski cells, suggesting a less compact Xi structure in these cells [15]. A recent study has also shown that deletion of CTCF sites causes disruption of TADs and spreading of euchromatin into heterochromatin [46]. Similarly, deletion of CTCF sites at the boundary of a Polycomb repressed domain results in transcriptional activation of genes in that domain [47].

Similar to our findings at escape genes, imprinted loci also show more contacts on the expressed allele, which probably reflects interactions between promoters and enhancers facilitated by allelic CTCF binding [3, 6, 7]. We discovered that the contacts are more frequent at paternally expressed genes (especially those on chromosome 7) than at maternally expressed genes. The cause of this bias is unclear and whether reported differences in the repeat (SINE) content and/or DNA sequence (GC content) of paternally and maternally expressed imprinted genes play a role is unknown [48, 49].

## Conclusions

Our 3D structure analysis of the mouse Xi reveals a bipartite structure. Two superdomains of frequent long-range contacts are separated by a hinge region that is partially conserved in human. The hinge region, which contains *Dxz4* and the minisatellite *Ds-TR*, represents a nucleolus-associated domain that may help target the Xi to the nucleolus. Together with CTCF and PolII binding, expressed escape genes tend to be located at the periphery of the Xi. In addition, analyses of genes that escape X inactivation and of imprinted genes indicate that expressed genes/alleles have more specific contacts compared with silenced genes/alleles.

## Materials and methods

### Tissues and cell lines

The Patski fibroblast line in which the Xi is from BL6 and the Xa from *M. spretus* was originally derived from embryonic kidney [27]. The presence of normal X chromosomes was verified by karyotyping. Whole brain was collected from female F1 adult mice obtained by mating *spretus* males (Jackson Labs) with females that carry an *Xist* mutation (B6.Cg-Xist<tm5Sado>) [50], in which there is complete skewing of inactivation of the *spretus* X. Liver specimens were collected from male and female BL6 adult mice [32]. Female MEFs [26] were cultured in standard complete medium. Primary neuron cultures were established on poly-lysine-coated coverslips from the hippocampus dissected from 0–2-day-old BL6 mouse pups.

### RNA-FISH, DNA-FISH, and immunostaining

RNA-FISH using a 10 kb *Xist* cDNA plasmid (pXho, which contains most of exon 1 of *Xist*) [51], and DNA-FISH for *Dxz4* (BAC clone RP23-299L1 from BACPAC) were done as described [32]. A whole mouse X chromosome painting probe (XMP X green from MetaSystems) was used for DNA-FISH together with *Dxz4* followed by *Xist* RNA-FISH using a standard protocol.

### ChIP-chip

ChIP-chip using an antibody for nucleophosmin (Abcam) was done as described [32]. Nimblescan software (Nimblegen Roche) was used to search for significant enrichment regions using a 500 bp sliding window. Enriched regions with a false discovery rate score less than 0.05 were considered as significant binding peaks.

### DNase Hi-C and in situ DNase Hi-C

DNAse Hi-C was done on mouse F1 brain and Patski cells using a previously published method [23]. In situ DNase Hi-C is described below.

#### Preparation of crosslinked cells

The whole brain from one F1 hybrid mouse was isolated and homogenized in 1× phosphate-buffered saline (PBS) with protease inhibitors followed by crosslinking with 1.5 % formaldehyde as described previously [25]. For Patski cells, one million cells were crosslinked in T-75 flasks with 1 % formaldehyde for 10 min followed by quenching with 125 mM glycine. Cells were scraped, washed in 1× PBS (Gibco), pelleted, and snap frozen in liquid nitrogen.

#### Chromatin digestion

Cell pellets containing approximately one million crosslinked cells were resuspended in cold lysis buffer (10 mM Tris–HCl pH 8.0, 10 mM NaCl, 0.2 % NP-40) and incubated on ice for 10 min. Nuclei were pelleted at 2500g for 60 s, resuspended in 100 μL of 0.5× DNase I digestion buffer [0.5× DNase I digestion buffer (Thermo), 0.5 mM MnCl_2_] containing 0.2 % SDS, and incubated at 37 °C for 30 min. An equal volume of 0.5× DNase I digestion buffer containing 2 % Triton X-100 and 4 U RNase A (Thermo) was added and incubation at 37 °C was continued for 10 min. Then, 1.5 U DNase I (Thermo) was added and digestion carried out at room temperature for 4 min. DNase I digestion was stopped by adding 40 μL of 6× Stop Solution (125 mM EDTA, 2.5 % SDS), followed by centrifugation at 2500g for 60 s. Nuclei were resuspended in 150 μL nuclease-free H_2_O (Ambion), and purified with two volumes (300 μL) of AMPure XP SPRI magnetic beads (Beckman Coulter). The resulting mixture was well mixed, incubated at room temperature for 5 min, collected via DynaMag-Spin magnet (Invitrogen), washed twice with 80 % ethanol, and air dried for 2 min.

#### Chromatin end-repair and dA-tailing

The purified bead-nuclei pellet was resuspended in 200 μL 1× T4 DNA Ligase Buffer (New England Biolabs) containing 0.25 mM dNTPs, 0.075 U/μl T4 DNA polymerase (Thermo) and 0.15 U/μl Klenow Fragment (Thermo), and incubated at room temperature for 1 h. The end-repair reaction was stopped by adding 5 μl of 10 % SDS. The bead-nuclei mixture was pelleted at 2500 g for 60 s, resuspended in 200 μl 1× NEB buffer 2 (New England Biolabs) containing 0.5mM dATP, 1 % Triton X-100 and 0.375 U/μl Klenow (exo-) (Thermo), and incubated at 37 °C for 1 h. dA-tailing reaction was stopped by adding 5 μl of 10 % SDS.

#### Bridge adaptor ligation

The bead-nuclei mixture was again pelleted at 2500g for 60 s, and resuspended in 30 μL H_2_O, 20 μL biotinylated bridge-adaptor (see Ma et al [23] for sequences and adaptor preparation), 20 μL blunt bridge-adaptor, 10 μL 10× T4 DNA Ligase Buffer with ATP, 10 μL polyethylene glycol (PEG)-4000 (Thermo), 5 μL 10 % Triton-X100, and 5 μL T4 DNA ligase (5 U/μL; Thermo). This mixture was incubated at 16 °C overnight to ligate T-tailed biotinylated bridge adapters to the termini of A-tailed, digested chromatin. Following incubation, the reaction was stopped by adding 5 μL 10 % SDS. The bead-nuclei mixture was then pelleted at 2500g for 60 s and resuspended in 300 μL H_2_O. To remove excess unligated adapter, 250 μL 20 % PEG in 2.5 M NaCl was added to the mixture, which was incubated at room temperature for 5 min, collected via DynaMag, and washed once with 80 % ethanol. Beads were then resuspended in 200 μL H_2_O and purified further using 0.8 volumes of 20 % PEG in 2.5 M NaCl as above, to further remove unligated adaptors.

#### Adaptor phosphorylation and proximity ligation

The air-dried bead-nuclei mixture was resuspended in 100 μL 1× T4 DNA Ligase Buffer with ATP containing 1 U/μL T4 Polynucleotide Kinase (PNK) (Thermo), and incubated at 37 °C for 1 h to phosphorylate ligated bridge adaptors. Following incubation, 90 μL 10× T4 DNA Ligase Buffer with ATP, 6 μL T4 DNA ligase (5 U/μL; Thermo), and 804 μL of H_2_O were added to the reaction mix. In situ proximity ligation was then carried out at room temperature for 4 h.

#### Reversal of crosslinking and purification of DNA

Following proximity ligation, bead-nuclei complexes were pelleted at 2500g for 60 s. Pellets were resuspended in 400 μL 1× NEBuffer #2, 40 μL 10 % SDS, and 40 μL Proteinase K (20 mg/ml; Thermo). This mixture was incubated overnight at 60 °C to reverse crosslinks and liberate ligated DNA. After incubation, DNA was precipitated by adding 3 μL GlycoBlue (Ambion), 50 μL 3 M sodium acetate pH 5.2, and 550 μL isopropanol and incubating the mixture at −80 °C for 2 h prior to centrifugation for 30 min at 15,000 rpm at 4 °C. The resulting bead-DNA pellet was resuspended in 100 μL H_2_O, then purified further using 100 μL AMPure XP beads, which were collected and washed as above. DNA was eluted using 100 μL H_2_O. Typical yields for experiments were 3–5 μg DNA per one million cells.

#### Sequencing library preparation

DNA (1.5–2.5 μg) was used for sequencing library preparation. End-repair was carried out by mixing 1.5–2.5 μg DNA in 170 μL H_2_O with 20 μL 10× End-repair reaction buffer (Thermo) and 10 μL Fast DNA End Repair Enzyme Mix (Thermo), and incubating the resulting mixture at 18 °C for 10 min. DNA was then purified using one volume (200 μL) AMPure XP beads, which were incubated, washed, and air-dried as above, then resuspended (including beads) in 50 μL 1× NEBuffer #2 containing 0.6 mM dATP and 12.5 U Klenow (exo-). This bead-enzyme mixture was then incubated at 37 °C for 30 min, after which 5 μL 10 % SDS was added to stop the dA-tailing reaction. The dA-tailed DNA-beads mixture was purified further by adding 1.6 volumes 20 % PEG in 2.5 M NaCl to the reaction. This mixture was incubated for 5 min, precipitated via DynaMag, washed twice with 80 % ethanol, air-dried, and resuspended in 50 μL 1× Rapid Ligation Buffer (Thermo) containing 5 μL 10× TruSeq Adapter (Illumina) and 20 U T4 DNA ligase. This mixture was incubated at room temperature for 1 h or at 16 °C overnight to ligate sequencing adapters, followed by quenching with 5 μL 10 % SDS. The ligation mixture was then brought to 200 μL with H_2_O and purified by adding 1 volume (200 μL) 20 % PEG in 2.5 M NaCl, immobilizing, washing, and air-drying beads as above. After air-drying, beads were resuspended in 200 μL H_2_O and purified further using 0.8 volumes of 20 % PEG in 2.5 M NaCl as above, to further remove unligated sequencing adaptors. DNA was eluted off of air-dried beads in 100 μL H_2_O, then pulled down with 30 μL MyOne C1 beads (Life Technologies) that had been washed and resuspended in 100 μL 2× Bind and Wash buffer (10 mM Tris–HCl pH 8.0, 1 mM EDTA, 2 M NaCl). Streptavidin pull-down was carried out for 20 min at room temperature with rotation. Immobilized DNA was precipitated via DynaMag, washed once with 600 μL 0.5× Bind and Wash buffer mixed with 0.5× TE lysis buffer (25 mM Tris–HCl, 0.5 mM EDTA, 0.5 % SDS), once with 600 μL 1× Bind and Wash buffer, once with 600 μL 1× NEBuffer #2, once with 600 μL Buffer EB (10 mM Tris–HCl pH 8.5), and resuspended in 20 μL Buffer EB. Libraries were then amplified for sequencing using 2× Robust Master Mix (KAPA), 10× PCR Primer Cocktail (Illumina) and half the volume of resuspended streptavidin beads, for 12 cycles, purified using 0.8× volumes of AMPure XP beads, then sequenced. Sequencing was carried out using Illumina HiSeq 2000 and NextSeq 500 instruments to generate paired-end 80 bp or paired-end 101 bp reads.

### NAD analysis

Nucleoli were isolated from fixed Patski cells using 1 % formadehyde using a modified method [34, 35]. In brief, two to three million cells were fixed for 10 min at room temperature and quenched using 0.125 M glycine. The fixed cells were resuspended in 1 ml of high magnesium buffer (10 mM HEPES, 0.35 M sucrose, 12 mM MgCl_2_ plus protease inhibitors) and sonicated for six rounds of 10-s bursts (full power) using a Misonix Sonicator3000. The dirty nucleoli preparation was centrifuged for 30 s at 15,000g and resuspended in 0.5 ml low magnesium buffer (10 mM HEPES, 0.88 M sucrose, 1 mM MgCl_2_ plus protease inhibitors), which was sonicated one more time with a 10-s burst (full power) and centrifuged again. The nucleoli pellet was used for DNA extraction and qPCR. Release of nucleoli was monitored by microscopy after immunostaining of the preparation with nucleophosim antibody (Abcam).

### Quantitative PCR

qPCR was performed using a SYBR green system as described before [32]. The primers used are listed in Additional file 9.

### Computational analyses

#### Mapping and filtering of sequence reads

We sequenced the DNase Hi-C libraries using paired-end reads 150 bp in length and the in situ DNase Hi-C libraries using paired-end reads 80 bp in length. We performed an exhaustive search and cleaning of the Illumina primer and adaptor sequences in the full-length reads and extracted the remaining read fragments of various lengths from 25 to 80 bp using an in-house script, as described in [23]. We then mapped each end of these cleaned paired-end reads separately to the BL6 genome using the NCBI build v37/mm9 reference genome assembly obtained from the UCSC Genome Browser [52] and the pseudo-*spretus* genome using BWA/v.0.5.9 [53]. The pseudo-*spretus* genome was assembled by substituting available SNPs (from Sanger Institute, SNP database Nov/2011 version) into the BL6 reference genome, as described in [15]. We retained only the reads that mapped uniquely, allowing at most three mismatches and requiring a mapping score MAPQ ≥30 to either the BL6 genome or the pseudo-*spretus* genome, for further analyses.

#### Allele-specific contact maps

Using heterozygous SNPs between the BL6 genome and the pseudo-*spretus* genome, we segregated all high-quality uniquely mapped reads (MAPQ ≥30) into three categories: (1) BL6-SNP reads containing only BL6-specific SNP(s); (2) *spretus*-SNP reads containing only *spretus*-specific SNP(s); (3) reads that do not contain valid SNPs. We refer to both BL6-SNP reads and *spretus*-SNP reads as “allele-specific reads”, and reads that do not contain valid SNPs as “allele-uncertain reads”. Furthermore, to eliminate the bias due to the PCR duplication step, we removed redundant paired-end reads. We define two reads as redundant if both ends of the reads are mapped to identical locations in the same genome assembly.

After PCR duplicate removal, we generated allele-specific whole-genome contact maps at 1 Mb, 100 kb and 40 kb resolutions. To do so, we partitioned the genome into non-overlapping bins and counted the number of allele-specific contacts (i.e., uniquely mapped paired-end reads) observed between each pair of bins. The dimension of the resulting contact map is the total number of bins in the genome, and entry (*i*, *j*) is the contact count between bins *i* and *j*. Specifically, in the allele-specific contact map of the Xa, ${C}_{i_1,{j}_1}$ denotes the contact counts between bins *i* and *j* on the Xa. Whereas in the allele-specific contact map of the Xi, ${C}_{i_0,{j}_0}$ denotes the contact counts between bins *i* and *j* on the Xi.

#### Inference of allele-uncertain reads

Using a similar approach to previous methods [54, 55], we model the contact frequencies between genomic loci pair as a binomial distribution *X*_*i*,*j*_ ~ *Binomial*(*M*, *p*_*i*,*j*_), where *M* is the total number of observed contacts (high-quality uniquely mapped and non-redundant paired-end reads) in a given (in situ) DNase Hi-C experiment. Since *M* is large and *p*_*i*,*j*_ is very small, we approximate the binomial distribution by a Poisson distribution *X*_*i*,*j*_ ~ *Poisson*(*λ*_*i*,*j*_), where *λ*_*i*,*j*_ = *Mp*_*i*,*j*_. Adapting to the diploid genome, we assume the observed allele-specific chromatin contact counts follow the Poisson model:

$$ {X}_{i_{\odot },{j}_{\otimes }}\sim Poisson\left({\lambda}_{i_{\odot },{j}_{\otimes }}\right), $$

where *i*_⊙_ ∈ {*i*_0_, *i*_1_} and *j*_⊗_ ∈ {*j*_0_, *j*_1_}, ${\uplambda}_{{\mathrm{i}}_{\odot },{\mathrm{j}}_{\otimes }}$ is the expected allele-specific contact counts between loci pair *i*_⊙_ and *j*_⊗_. Furthermore, we assume the Poisson parameter ${\uplambda}_{{\mathrm{i}}_{\odot },{\mathrm{j}}_{\otimes }}$ follows a gamma prior distribution:

$$ {\lambda}_{i_{\odot },{j}_{\otimes }}\sim Gamma\kern0.5em \left({\alpha}_{G\left({i}_{\odot },{j}_{\otimes}\right)},{\beta}_{G\left({i}_{\odot },{j}_{\otimes}\right)}\right). $$

The hyper-parameters ***α*** and ***β*** depend on *G*(*i*_⊙_, *j*_⊗_), which is the genomic group assignment of loci pair *i*_⊙_ and *j*_⊗_ for accommodating the systematic differences of expected contacting frequencies between intrachromosomal contacts and interchromosomal contacts.

Based on the observations that the intrachromosomal contact frequency decreases as the genomic distance increases and interchromosomal contacts are rare, we model that the hyper-parameter ***α*** and ***β*** are shared across intrachromosomal contacts between similar genomic distance as well as interchromosomal contacts between two separate chromosomes. Thus, we have:

$$ G\left({i}_{\odot },{j}_{\otimes}\right)=\left\{\begin{array}{c}\hfill {g}_{k_{\odot },d\left({i}_{\odot },{j}_{\otimes}\right)}\kern1em \mathrm{if}\ chr\left({i}_{\odot}\right)={k}_{\odot }={k}_{\otimes }=chr\left({j}_{\otimes}\right)\hfill \\ {}\hfill {g}_{k_{\odot },{k}_{\otimes }}\kern2.95em \mathrm{if}\ chr\left({i}_{\odot}\right)={k}_{\odot}\ne {k}_{\otimes }=chr\left({j}_{\otimes}\right)\hfill \end{array}.\right. $$

That is, for intrachromosomal contacts, all loci pairs *i*_⊙_ and *j*_⊗_ on the same chromosome *k*_⊙_ and with the same genomic distance *d*(*i*_⊙_, *j*_⊗_) (binned at given resolution) share the same gamma prior hyper-parameters. On the other hand, for interchromosomal contacts, all loci pairs from the same pair of chromosomes *k*_⊙_ and *k*_⊗_ share the same gamma prior hyper-parameters.

Posterior mean estimates of allele-specific contact frequencies ${\uplambda}_{i_{\odot },{j}_{\otimes }}$ and the hyper-parameters ***α*** and ***β*** are obtained via the expectation-maximization (EM) algorithm [56]: (1) we assign allele-uncertain reads to the allele-specific contact maps based on the estimates of allele-specific reads; (2) we estimate the hyper-parameters ***α*** and ***β*** using the empirical Bayes approach and calculate the posterior mean estimates of ${\uplambda}_{i_{\odot },{j}_{\otimes }}$; (3) we re-assign allele-uncertain reads based on the current estimation $\overline{\uplambda_{i_{\odot },{j}_{\otimes }}}$ and update the inferred allele-specific contact maps. We repeat steps 2–3 until convergence. For 1 Mb resolution analysis, we use contact maps containing only allele-specific reads, while for finer-resolution at 100 kb or 40 kb, we use the inferred allele-specific contact maps in our analyses.

#### Normalization

We normalized the allele-specific contact maps obtained from DNase Hi-C and in situ DNase Hi-C data using an iterative correction method [57]. Here we only used intrachromosomal contacts to normalize the allele-specific contacts. This is based on the observation that interchromosomal (including inter-homologous) contacts are rare. We first preprocessed the allele-specific intrachromosomal contact maps at 1 Mb, 100 kb or 40 kb resolution by setting the entries that may be dominated by self-ligation products to 0. These entries are the diagonal, super-diagonal (+1 off-diagonal) and sub-diagonal (−1 off-diagonal) contact counts. In addition, we excluded bins with the lowest 2 % read coverage. Lastly, we applied the iterative correction procedure on each preprocessed intrachromosomal contact map separately to obtain a normalized contact map with near-equal row and column sums.

#### Topological domain calling

We identified topological domains using a previously described hidden Markov model-based software tool [21]. We applied the topological domain calling on normalized diploid contact maps at 40 kb resolution. As in previous work [21], we classified the regions between the topological domains either as “domain boundaries” (≤400 kb) or “unorganized chromatin” (>400 kb).

#### Assigning statistical significance to normalized contact maps

To obtain a set of high-confidence contacts, we subjected the diploid contact maps at 40 kb resolution to a statistical confidence estimation procedure, fit-hi-c [54]. The procedure accounts for the effect of genomic distance on the intrachromosomal contact probability by fitting a smoothing spline. We then accounted for biases using the normalization procedure described above. Finally, we applied multiple hypothesis testing to compute q values, which are used to filter contacts at a desired false discovery rate at 0.05.

#### Assessing superdomain contact density on Xi

To measure density of the two superdomains on the Xi, we calculated the ratio of intra- versus inter-superdomain contact frequencies, called the bipartite index (BI), as follows:

$$ \frac{\frac{{\displaystyle {\sum}_{i=1}^h}{\displaystyle {\sum}_{j=1}^h}{C}_{i,j}}{h^2}+\frac{{\displaystyle {\sum}_{i=h+1}^n}{\displaystyle {\sum}_{j=h+1}^n}{C}_{i,j}}{{\left(n-h\right)}^2}}{2\frac{{\displaystyle {\sum}_{i=1}^h}{\displaystyle {\sum}_{j=h+1}^n}{C}_{i,j}}{h\left(n-h\right)}}, $$

where *C*_*i*,*j*_ is the allele-specific contact counts for the X chromosome of interest, *n* is the total number of bins in the chromosomal contact map, and *h* is the index of superdomain boundary (that is, the hinge region). We calculated the BIs for Xi and Xa in both F1 brain and Patski datasets. A higher BI value represents more condensed packaging of the chromatins within the two superdomains. To measure the significance of the bipartite structure of the Xi, we randomly shifted the superdomain boundary to estimate the null distribution of the bipartite index. We then used the one-sided Z-test to calculate the *p* value for the observed BI at the hinge region (Table 1).

#### Comparison between human and mouse X chromosome contact profiles

To construct the synteny map between human and mouse X chromosomes, we used the UCSC liftOver utility [58] to convert the mouse/mm9 coordinates of all refSeq genes on chromosome X [59] to the human/hg19 coordinates. We only used mouse X-linked genes that have a homologous human X-linked counterpart to plot the synteny map in Fig. 2. We used the same tool to convert the human/hg19 coordinates of the 27 superloops reported on the human X chromosome [6] to mouse/mm9 coordinates.

#### Inference of the 3D structure of X chromosomes

We inferred the 3D structure of the Xa and Xi chromosomes, separately, using the Pastis software [60]. Each X chromosome is modeled as a series of beads on a string, spaced 1 Mb apart. We denote by *X* = (*x*_1_, *x*_2_, ⋯, *x*_*n*_) ∈ ℝ^3^ the coordinate matrix of the structure, where *n* denotes the total number of beads on the chromosome (*n* = 167 for the mouse X chromosome), and *x*_*i*_ ∈ ℝ^3^ represents the 3D coordinates of the *i*-th bead.

The Pastis model assumes that the observed contact counts *C*_*i*,*j*_ between beads *i* and *j* follows a Poisson distribution, where the Poisson parameter of *C*_*i*,*j*_ is a decreasing function of *d*_*ij*_(***X***) of the form *βd*_*ij*_(***X***)^*α*^, and *d*_*ij*_(***X***) = ||*x*_*i*_ − *x*_*j*_|| is the Euclidean distance between the beads *i* and *j*. Therefore, the problem of 3D structure inference is formulated as the following optimization problem:

$$ { \max}_{\beta,\ \boldsymbol{X}}\mathrm{\mathcal{L}}\left(\boldsymbol{X}\right)={\displaystyle \sum_{1\le i<j\le n}}{C}_{i,j}\alpha \log {d}_{ij}\left(\boldsymbol{X}\right)+{C}_{i,j} \log \beta -\beta {d}_{ij}{\left(\boldsymbol{X}\right)}^{\alpha }. $$

Here we set *α* = −3 and optimize the structure and *β* using IPOPT, an interior point filter algorithm [61].

#### Enrichment of escape genes at X chromosome periphery

To measure the 3D positional preference of escape genes with regards to the X chromosome periphery, we calculated the radial distances of escape genes to the chromosome center and superdomain centers as described below.

The center of the X chromosome is located at the origin, that is, ${\displaystyle {\sum}_{i=1}^n}{x}_i=\left(0,0,0\right)$*.* For each escape gene *g*, the distance of gene *g* to the chromosome center is *d*_*g*_ = ||*x*_*k*_||, where *k* is the index of the bin that is closest to the middle point of the gene. In addition, given the observation that Xi forms a bipartite structure and the hinge region is located at locus *h*, we computed the centers of the two superdomains as ${c}_1=\frac{1}{h}{\displaystyle {\sum}_{i=1}^{h-1}}{x}_i$ and ${c}_2=\frac{1}{n-h}{\displaystyle {\sum}_{i=h+1}^n}{x}_i$, respectively. Then the distances of escape gene *g* to the superdomain centers are calculated as ||*x*_*k*_ − *c*_1_|| and ||*x*_*k*_ − *c*_2_||.

To test the enrichment of escape genes at the chromosome periphery or at the superdomain periphery, we randomly sampled 100 X-linked genes to estimate the expected distance to the chromosome or superdomain center and then evaluated the significance of observed distances of escape genes using a Z-test.

#### Correlation between one-dimensional genomic features and 3D structure

To investigate the spatial distribution of genetic and epigenetic features of the X chromosomes in the 3D nucleus space, we performed the following analyses for three different genetic and epigenetic features on both Xa and Xi: (1) allele-specific CTCF binding peaks in brain and Patski cells [15]; (2) allele-specific PolII peaks in brain and Patski cells [15]; (3) L1 elements (downloaded from UCSC Genome Browser).

First, we asked whether feature-rich regions are enriched at the chromosome or superdomain periphery. For each non-overlapping 1 Mb bin *i* along the X chromosome, we computed the density of the given feature at bin *i*. Then we visualized the feature density as a function of the radial distance to the chromosome or superdomain center using a boxplot. In addition, we asked whether feature-rich regions tend to locate near the periphery or interior of the chromosome or superdomains. We define feature-rich regions as bins that fall within the top 25 % in terms of feature density, and we define feature-poor regions as bins within the bottom 25 % of feature density. Then we investigated the empirical cumulative density function of the distance to the chromosome or superdomain center for the feature-rich and feature-poor regions, and then evaluated the difference between the two distributions using one-side Wilcoxon rank-sum test.

#### Analysis of contacts at escape genes and imprinted genes

For this analysis we used allele-specific Hi-C contact maps at 40 kb resolution. Starting with a list of imprinted genes from a published study [36] we filtered the list to include only genes where our RNA-seq data indicated biased expression towards the putatively expressed allele based on a binomial test. The binomial parameter was derived from the set of all autosomal genes by taking the average ratio of maternal to paternal read counts. Multiple testing correction was performed with the Benjamini-Hochberg procedure, and a q value of less than 0.05 was considered significant. For the list of genes that escape XCI we used a previously established list [15] from which we selected those with an average PolII SNP read count of ≥5 in 100 bp intervals at 0.5 kb upstream and downstream of the transcription start site. For the list of autosomal and X-linked genes we downloaded the UCSC knownGenes table, retaining entries that were also listed in Ensembl. We excluded genes that overlap within the same Hi-C window (40 kb resolution) and any of the genes in our lists of imprinted genes or X escape genes. In cases where multiple genes in the remaining set fell within exactly the same Hi-C window, we included only one gene in the background distribution. Finally, we also eliminated any genes for which no contacts were observed in the bulk Hi-C contact map. Background distributions were separated for autosomal and X-linked genes.

For each gene across our imprinted, X escape, and background sets, we performed a virtual 4C analysis, where we extracted one or more columns from the paternal and maternal allele-specific contact maps. The contacts in these columns were summed for each allele prior to calculating $log10\left(\frac{\mathrm{maternal}\;\mathrm{contact}\;\mathrm{count}+1}{\mathrm{paternal}\;\mathrm{contact}\;\mathrm{count}+1}\right)$. Note that the +1 in the numerator and denominator acts as a pseudocount. For each imprinted gene set, we used a two-sided Kolmogorov-Smirnov test to test for significant deviation from the autosomal gene background distribution. The same test was performed for escape genes in comparison to all X-linked genes used to determine the background distribution. Similar significance values were obtained from alternative significance metrics such as a Wilcoxon rank sum test.

### Accession numbers

The RNA-seq, ChIP-chip, ChIP-seq, and DNase Hi-C data are available in the Gene Expression Omnibus (GEO) database, under the accession numbers GSE30761 and GSE59779 (subseries GSE68992).

### Ethics statement

For mice sacrificed, euthanasia was accomplished using two methods (carbon dioxide asphyxiation followed by cervical dislocation) as required by the University of Washington’s Office of Animal Welfare. Husbandry and all other procedures were approved by the University of Washington’s Office of Animal Welfare (Protocol 2254).

## Additional files

Additional file 1: Table S1. Summary of allele-specific contacts. (XLSX 10 kb) Additional file 2: Table S2. Summary of the number of contacts obtained by DNase Hi-C and in situ DNase Hi-C in relation to distance. (XLSX 9 kb) Additional file 3: Figure S1. Allelic differences between contact maps for the X chromosomes are not seen for homologous autosomes. Allelic intrachromosomal chromatin contact heatmaps of chromosome 1 homologs based on SNP reads at 1 Mb resolution obtained by DNase Hi-C and in situ DNase Hi-C in F1 brain and in Patski cells. Contact maps for chromosomes 1 appear remarkably similar between maternal (BL6) and paternal (*spretus*) chromosomes. See Fig. 1a for comparison with contacts maps obtained for the Xa and Xi, which demonstrate striking differences. (PDF 1178 kb) Additional file 4: Figure S2. TADs are more prominent on Xa versus Xi. Contact maps for the Xa and Xi at 100 kb resolution for chrX:98,500,00–103,499,999 in F1 brain (*top*) and Patski cells (*bottom*). (PDF 717 kb) Additional file 5: Figure S3. Mapping of the hinge region. Contact maps at the hinge region in F1 brain and Patski cells. Data are shown at a 100 kb resolution for a region of chrX:70,000,000–75,099,999 (*left*) and at a 40 kb resolution for chrX:70,000,000–75,039,999 (*right*). The estimated boundaries of the superdomains 1 and 2 (at 100 kb or 40 kb) are marked by *dotted red lines* and the hinge region located in between the superdomains contains the least number of contacts. (PDF 825 kb) Additional file 6: Figure S4. Reproducibility of Xi contact maps obtained by DNase Hi-C and in situ DNase Hi-C approaches in F1 brain and Patski cells. Allelic intrachromosomal chromatin contact heatmaps are shown for the mouse Xa and Xi based on SNP reads at 1 Mb resolution using DNase Hi-C and in situ DNase Hi-C to the same whole brain specimen, and for two independent biological replicates using in situ DNase Hi-C on Patski cells. These contact maps show remarkably similar features between replicates, between methods, and between in vitro and in vivo mouse hybrid systems. Note that XCI is reciprocal between F1 brain (*spretus* Xi) and Patski cells (BL6 Xi). (PDF 1598 kb) Additional file 7: Table S3. List of imprinted genes and genes that escape XCI in mouse F1 brain. (XLSX 12 kb) Additional file 8: Figure S5. Intrachromosomal contacts at *Kcnk9* and at imprinted genes, excluding those located on chromosome 7. **a** Significant contacts are detected at the maternally expressed gene *Kcnk9* on the maternal allele (*Mat*). Allelic mRNA-seq profiles show expression on the maternal allele at imprinted genes *Kcnk9*, *Trappc9*, *Chrac1* and *Ago2*, and on the paternal allele (*Pat*) at *Peg13*, in agreement with a previous study [62]*.* Allelic CTCF profiles show absence of binding to the differentially methylated region (*DMR*) on the maternal allele (*arrow*) presumably facilitating the formation of contacts between the *Kcnk9* promoter region and an unidentified distant enhancer, similar to the situation in human [62]. The needle plot of contact counts between a 40 kb window that overlaps *Kcnk9* (*grey bar*) and other regions shows more interactions on the maternal (*pink*) than the paternal allele (*blue*). Genes with maternal or paternal expression are colored *pink* or *blue*, non-imprinted genes *black*, and non-expressed genes *grey*, respectively. Contact regions showing significant allelic biases are marked by *asterisks*. **b** Distribution of maternal-to-paternal allelic contacts at autosomal genes and X-linked genes determined by DNase Hi-C at 40 kb resolution in F1 brain in which the paternal chromosomes are from *spretus*. **c** Violin plots show the distribution of maternal-to-paternal allelic contacts at maternally and paternally imprinted genes at 40 kb resolution in F1 brain, after removing genes located on chromosome 7, which changes the shape of the distribution, due to fewer genes showing a low maternal-to-paternal contact ratio (see also Fig. 7). *Dotted line* indicates median ratios of maternal-to-paternal contacts at autosomal genes. (PDF 1263 kb) Additional file 9: Table S4. List of primers. (XLS 20 kb)

## Abbreviations

3D: three-dimensional
BI: bipartite index
bp: base pair
ChIP: chromatin immunoprecipitation
FISH: fluorescence in situ hybridization
HAT: hypoxanthine-aminopterin-thymidine
L1: LINE1
LAD: lamina-associated domain
lncRNA: long non-coding RNA
MEF: mouse embryonic fibroblast
NAD: nucleolus-associated domain
PBS: phosphate-buffered saline
PEG: polyethylene glycol
PolII: RNA polymerase II phosphorylated at serine 5
qPCR: quantitative PCR
SNP: single nucleotide polymorphism
TAD: topologically associated domain
Xa: active X chromosome
XCI: X chromosome inactivation
Xi: inactive X chromosome
