# Supplementary figures and images for: Bipartite structure of the inactive mouse X chromosome
Source: Genome Biol. 2015 Aug 7;16(1):152. doi: 10.1186/s13059-015-0728-8 (PMC4539712; doi:10.1186/s13059-015-0728-8)

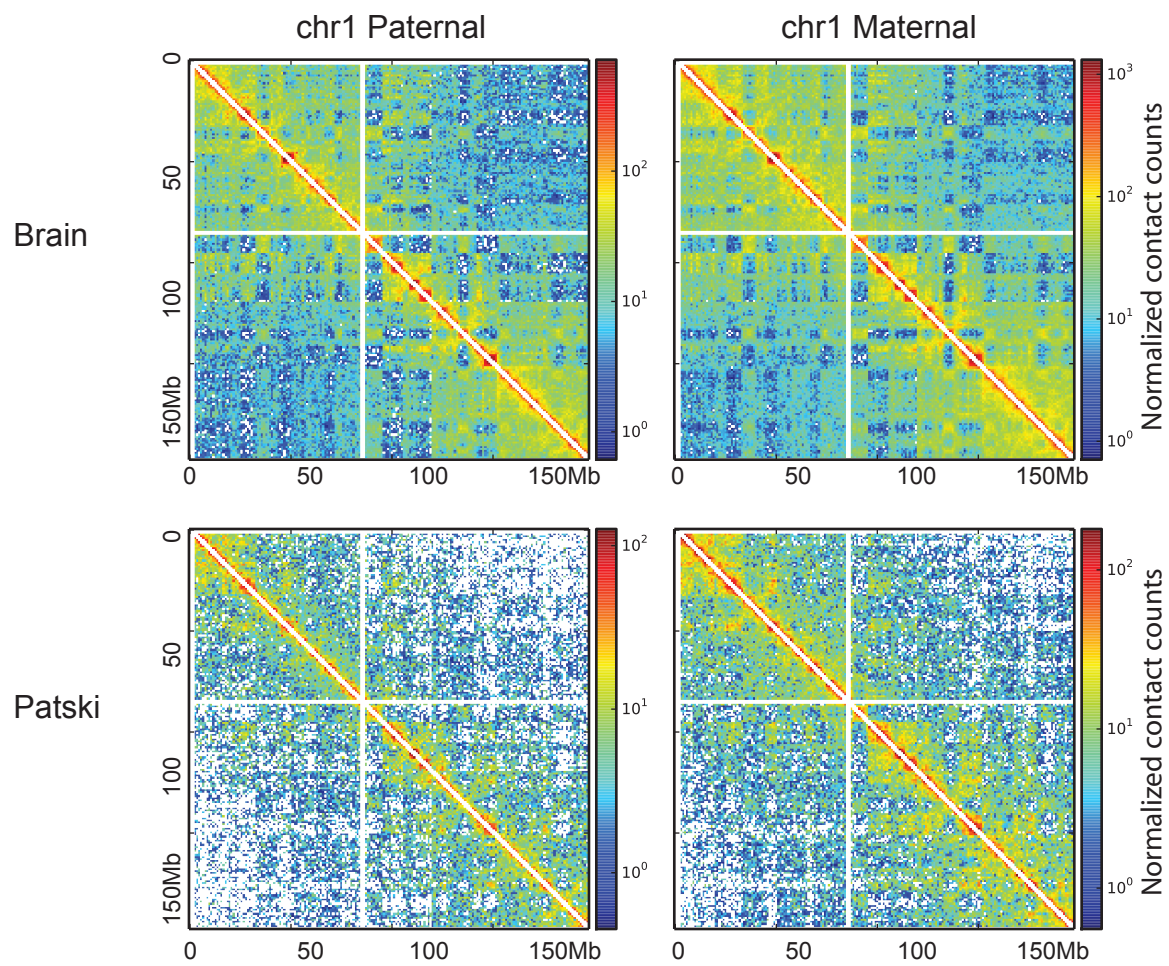

Supplement: Additional file 3: Figure S1. — Allelic differences between contact maps for the X chromosomes are not seen for homologous autosomes. Allelic intrachromosomal chromatin contact heatmaps of chromosome 1 homologs based on SNP reads at 1 Mb resolution obtained by DNase Hi-C and in situ DNase Hi-C in F1 brain and in Patski cells. Contact maps for chromosomes 1 appear remarkably similar between maternal (BL6) and paternal (spretus) chromosomes. See Fig. 1a for comparison with contacts maps obtained for the Xa and Xi, which demonstrate striking differences. (PDF 1178 kb) [file 13059_2015_728_MOESM3_ESM.pdf]

active X

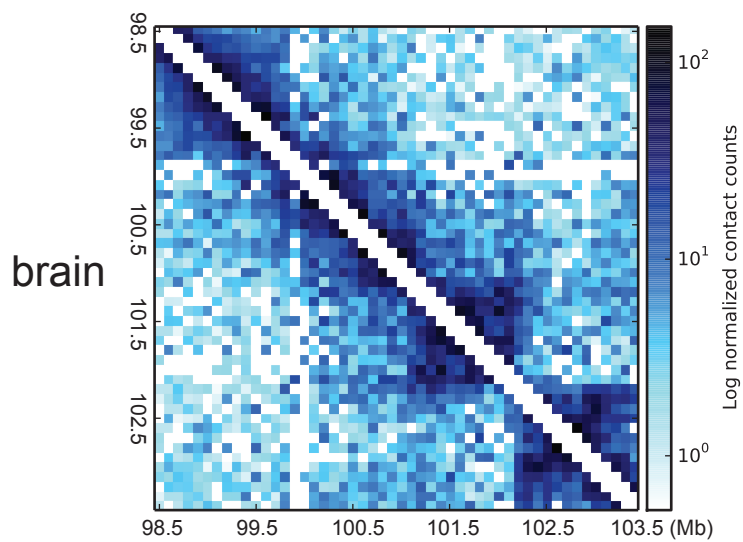

inactive X

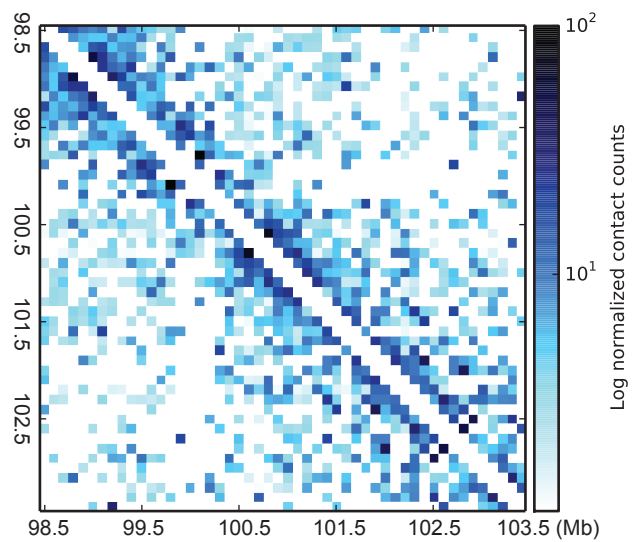

Patski

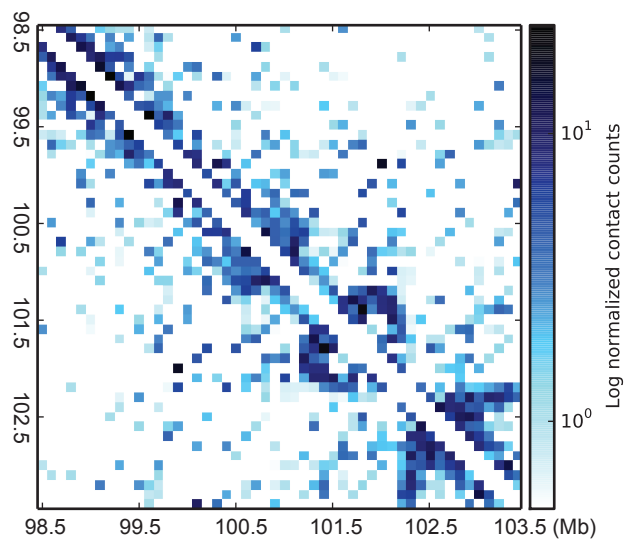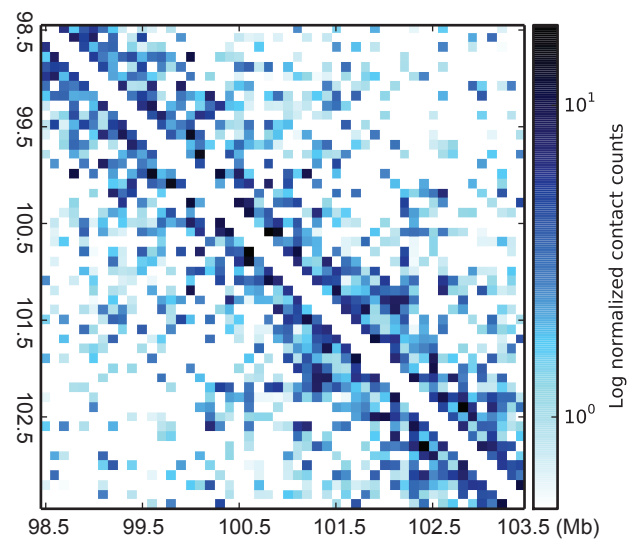

Supplement: Additional file 4: Figure S2. — TADs are more prominent on Xa versus Xi. Contact maps for the Xa and Xi at 100 kb resolution for chrX:98,500,00–103,499,999 in F1 brain (top) and Patski cells (bottom). (PDF 717 kb) [file 13059_2015_728_MOESM4_ESM.pdf]

100kb

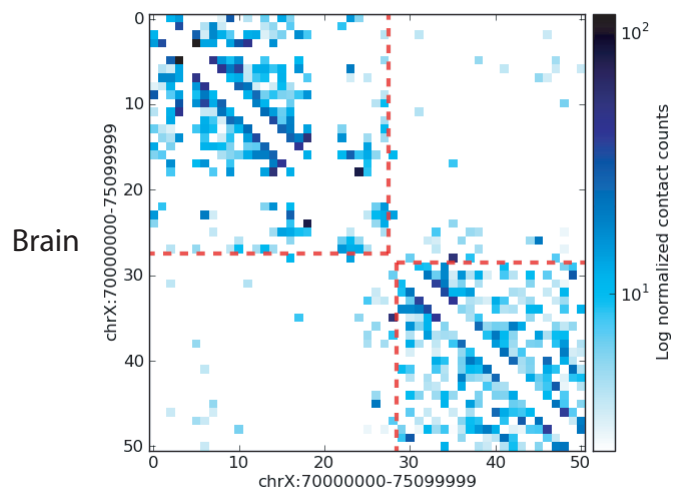

40kb

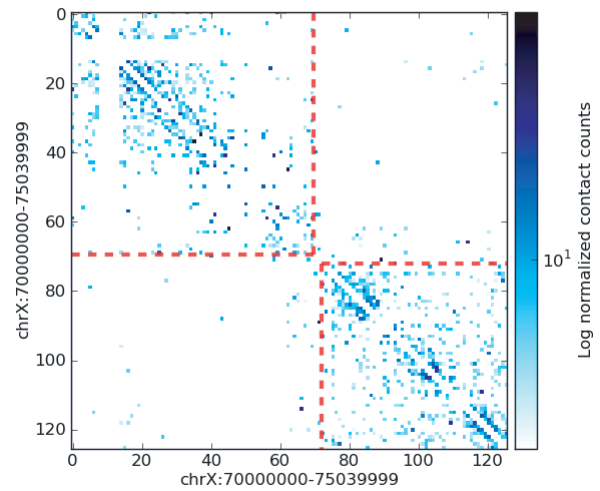

Patski

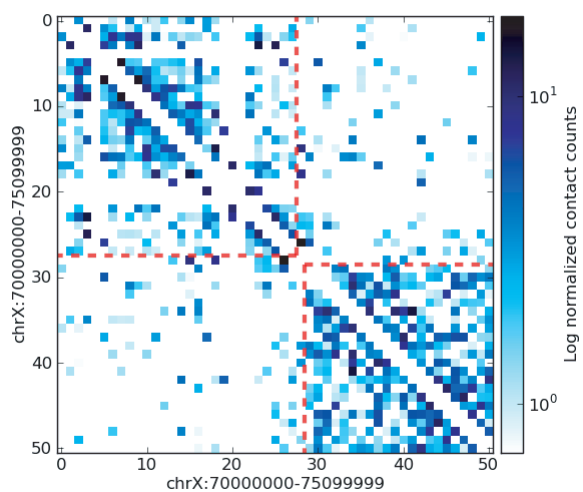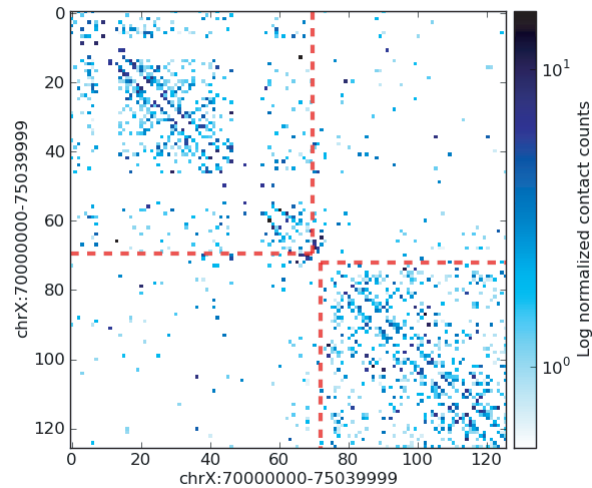

Supplement: Additional file 5: Figure S3. — Mapping of the hinge region. Contact maps at the hinge region in F1 brain and Patski cells. Data are shown at a 100 kb resolution for a region of chrX:70,000,000–75,099,999 (left) and at a 40 kb resolution for chrX:70,000,000–75,039,999 (right). The estimated boundaries of the superdomains 1 and 2 (at 100 kb or 40 kb) are marked by dotted red lines and the hinge region located in between the superdomains contains the least number of contacts. (PDF 825 kb) [file 13059_2015_728_MOESM5_ESM.pdf]

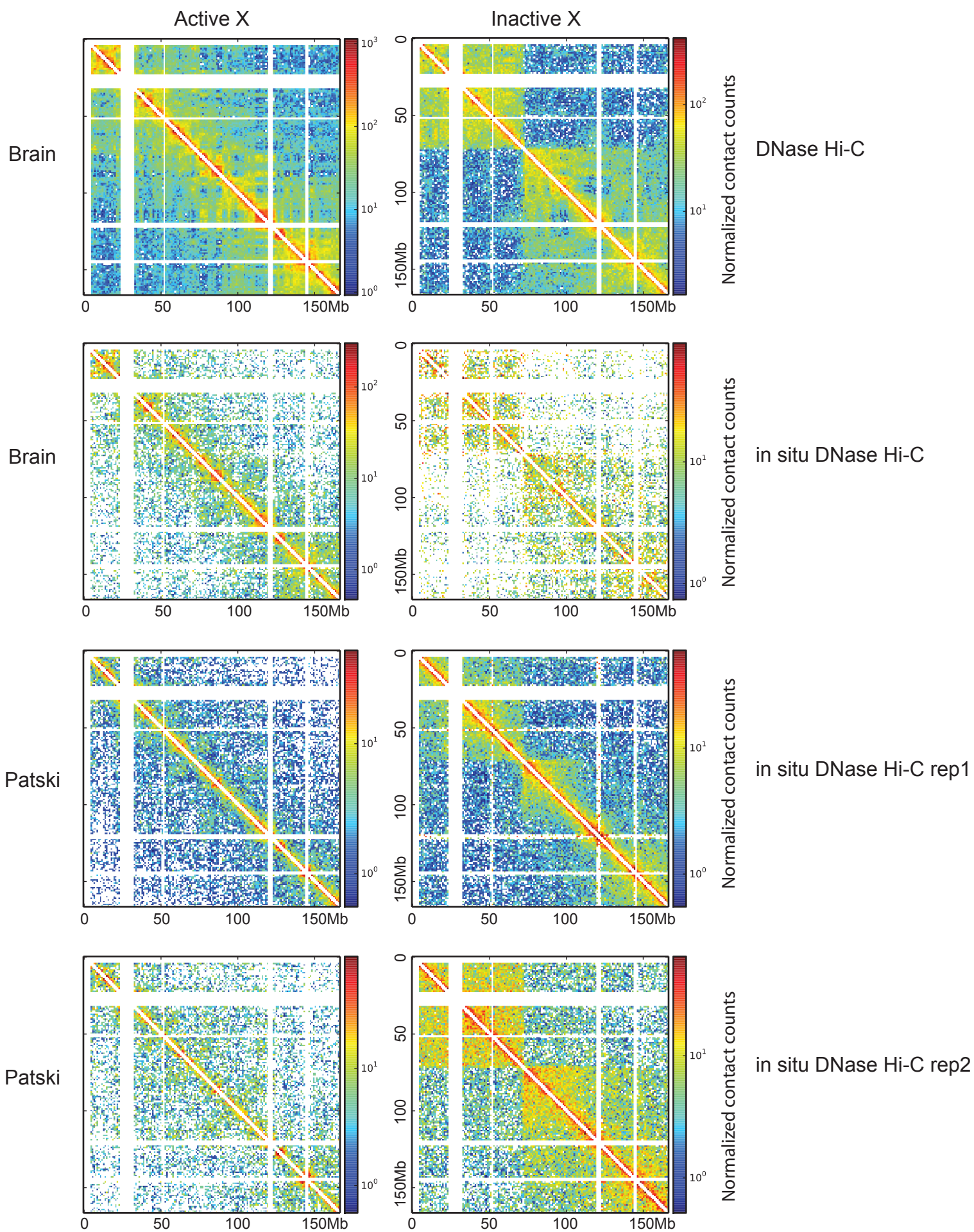

Supplement: Additional file 6: Figure S4. — Reproducibility of Xi contact maps obtained by DNase Hi-C and in situ DNase Hi-C approaches in F1 brain and Patski cells. Allelic intrachromosomal chromatin contact heatmaps are shown for the mouse Xa and Xi based on SNP reads at 1 Mb resolution using DNase Hi-C and in situ DNase Hi-C to the same whole brain specimen, and for two independent biological replicates using in situ DNase Hi-C on Patski cells. These contact maps show remarkably similar features between replicates, between methods, and between in vitro and in vivo mouse hybrid systems. Note that XCI is reciprocal between F1 brain (spretus Xi) and Patski cells (BL6 Xi). (PDF 1598 kb) [file 13059_2015_728_MOESM6_ESM.pdf]

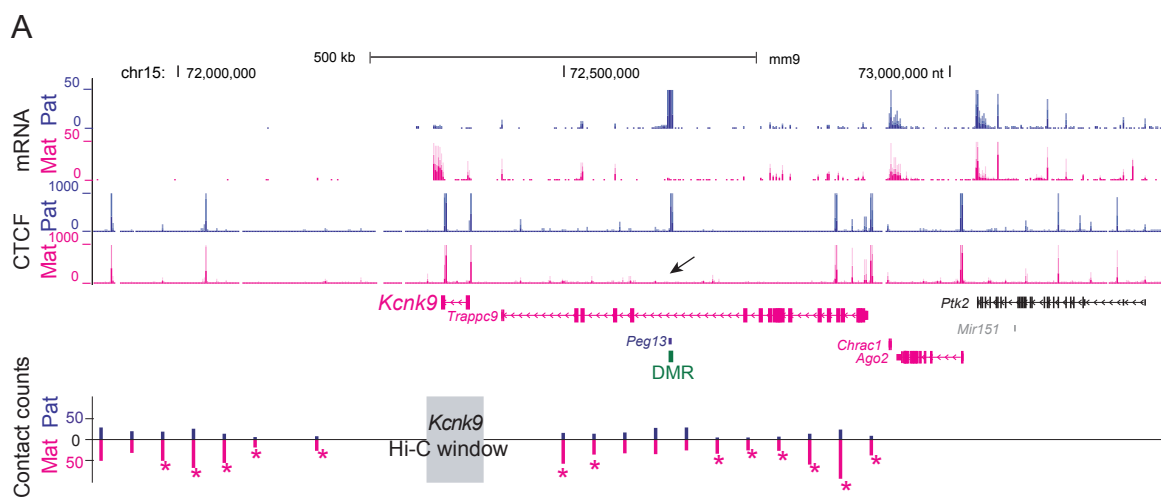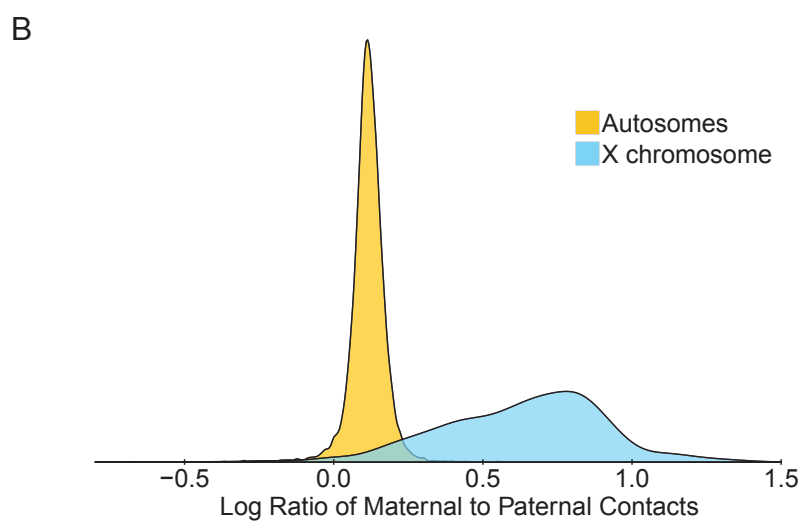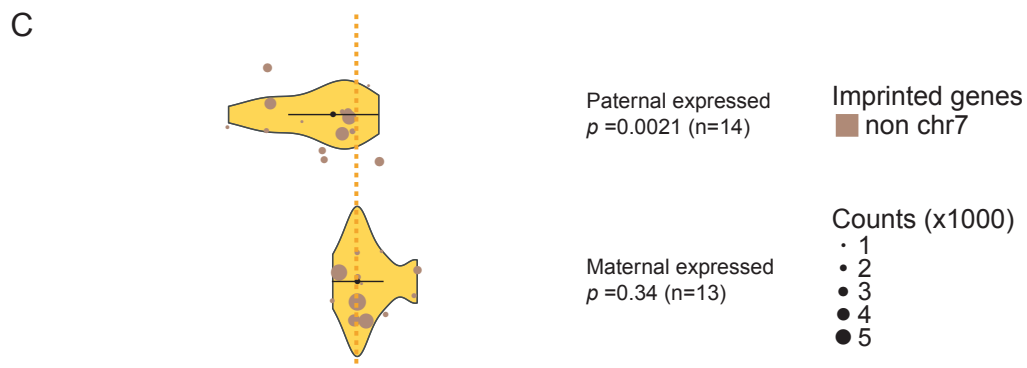

Supplement: Additional file 8: Figure S5. — Intrachromosomal contacts at Kcnk9 and at imprinted genes, excluding those located on chromosome 7. a Significant contacts are detected at the maternally expressed gene Kcnk9 on the maternal allele (Mat). Allelic mRNA-seq profiles show expression on the maternal allele at imprinted genes Kcnk9, Trappc9, Chrac1 and Ago2, and on the paternal allele (Pat) at Peg13, in agreement with a previous study [62]. Allelic CTCF profiles show absence of binding to the differentially methylated region (DMR) on the maternal allele (arrow) presumably facilitating the formation of contacts between the Kcnk9 promoter region and an unidentified distant enhancer, similar to the situation in human [62]. The needle plot of contact counts between a 40 kb window that overlaps Kcnk9 (grey bar) and other regions shows more interactions on the maternal (pink) than the paternal allele (blue). Genes with maternal or paternal expression are colored pink or blue, non-imprinted genes black, and non-expressed genes grey, respectively. Contact regions showing significant allelic biases are marked by asterisks. b Distribution of maternal-to-paternal allelic contacts at autosomal genes and X-linked genes determined by DNase Hi-C at 40 kb resolution in F1 brain in which the paternal chromosomes are from spretus. c Violin plots show the distribution of maternal-to-paternal allelic contacts at maternally and paternally imprinted genes at 40 kb resolution in F1 brain, after removing genes located on chromosome 7, which changes the shape of the distribution, due to fewer genes showing a low maternal-to-paternal contact ratio (see also Fig. 7). Dotted line indicates median ratios of maternal-to-paternal contacts at autosomal genes. (PDF 1263 kb) [file 13059_2015_728_MOESM8_ESM.pdf]
